# Current and future role of MRI in the diagnosis and prognosis of multiple sclerosis

**DOI:** 10.1016/j.lanepe.2024.100978

**Published:** 2024-08-22

**Authors:** Maria A. Rocca, Paolo Preziosa, Frederik Barkhof, Wallace Brownlee, Massimiliano Calabrese, Nicola De Stefano, Cristina Granziera, Stefan Ropele, Ahmed T. Toosy, Àngela Vidal-Jordana, Massimiliano Di Filippo, Massimo Filippi

**Affiliations:** a Neuroimaging Research Unit, Division of Neuroscience, IRCCS San Raffaele Scientific Institute, Milan, Italy; b Neurology Unit, IRCCS San Raffaele Scientific Institute, Milan, Italy; c Vita-Salute San Raffaele University, Milan, Italy; d Department of Radiology & Nuclear Medicine, Amsterdam UMC, Vrije Universiteit, Amsterdam, the Netherlands; e Queen Square Institute of Neurology and Centre for Medical Image Computing, University College London, London, UK; f Queen Square MS Centre, Department of Neuroinflammation, UCL Institute of Neurology, London, UK; g The Multiple Sclerosis Center of University Hospital of Verona, Department of Neurosciences and Biomedicine and Movement, Verona, Italy; h Department of Medicine, Surgery and Neuroscience, University of Siena, Siena, Italy; i Department of Neurology, University Hospital Basel and University of Basel, Basel, Switzerland; j Research Center for Clinical Neuroimmunology and Neuroscience Basel (RC2NB), University Hospital Basel and University of Basel, Basel, Switzerland; k Translational Imaging in Neurology (ThINk) Basel, Department of Biomedical Engineering, University Hospital Basel and University of Basel, Basel, Switzerland; l Department of Neurology, Medical University of Graz, Graz, Austria; m Servicio de Neurología, Centro de Esclerosis Múltiple de Catalunya (Cemcat), Hospital Universitari Vall d’Hebron, Universitat Autònoma de Barcelona, Barcelona, Spain; n Section of Neurology, Department of Medicine and Surgery, University of Perugia, Perugia, Italy; o Neurorehabilitation Unit, IRCCS San Raffaele Scientific Institute, Milan, Italy; p Neurophysiology Service, IRCCS San Raffaele Scientific Institute, Milan, Italy

**Keywords:** Multiple sclerosis, Magnetic resonance imaging, Diagnosis

## Abstract

In the majority of cases, multiple sclerosis (MS) is characterized by reversible episodes of neurological dysfunction, often followed by irreversible clinical disability. Accurate diagnostic criteria and prognostic markers are critical to enable early diagnosis and correctly identify patients with MS at increased risk of disease progression. The 2017 McDonald diagnostic criteria, which include magnetic resonance imaging (MRI) as a fundamental paraclinical tool, show high sensitivity and accuracy for the diagnosis of MS allowing early diagnosis and treatment. However, their inappropriate application, especially in the context of atypical clinical presentations, may increase the risk of misdiagnosis. To further improve the diagnostic process, novel imaging markers are emerging, but rigorous validation and standardization is still needed before they can be incorporated into clinical practice. This Series article discusses the current role of MRI in the diagnosis and prognosis of MS, while examining promising MRI markers, which could serve as reliable predictors of subsequent disease progression, helping to optimize the management of individual patients with MS. We also explore the potential of new technologies, such as artificial intelligence and automated quantification tools, to support clinicians in the management of patients. Yet, to ensure consistency and improvement in the use of MRI in MS diagnosis and patient follow-up, it is essential that standardized brain and spinal cord MRI protocols are applied, and that interpretation of results is performed by qualified (neuro)radiologists in all countries.

## Introduction

The diagnosis of multiple sclerosis (MS) requires evidence of a symptomatic demyelinating syndrome with objective neurologic signs, the evaluation of clinical and paraclinical findings to demonstrate a focal demyelinating pathology affecting at least two distinct central nervous system (CNS) areas (i.e., dissemination in space [DIS]) occurring at separate times (i.e., dissemination in time [DIT]),^1^ and the exclusion of alternative diagnoses.^2^, ^3^, ^4^ Magnetic resonance imaging (MRI) was formally included in the diagnostic algorithm for patients presenting with a clinically isolated syndrome (CIS) suggestive of MS in the 2001 McDonald criteria.^5^ These criteria, revised several times in the years up to the most recent revision in 2017,^1^ rely on the application of standardized MRI protocols^6^ to assess the number, size, and location of brain and spinal cord lesions typical of MS,^2^ thus allowing earlier MS diagnosis and to start treatment.

**Key messages**

• Magnetic resonance imaging (MRI) is crucial for diagnosing multiple sclerosis (MS) due to its ability to detect specific pathological processes with high accuracy.
• The 2017 McDonald diagnostic criteria accurately predict a second MS attack in patients with typical clinically isolated syndromes, even in pediatric patients, enabling early diagnosis and treatment. However, they should be used cautiously after ruling out other potential diagnoses.
• Emerging imaging markers like optic nerve involvement, cortical lesions, lesions with the central vein sign, and chronic active lesions may improve the accuracy of diagnostic criteria.
• Primary progressive MS is characterized by gradual progression, with diagnostic emphasis on cerebrospinal-fluid-specific oligoclonal bands and spinal cord lesions.
• Late-onset MS after 45–50 years may pose diagnostic challenges due to comorbidities and a more severe course.
• The number and location of white matter lesions in the brainstem and spinal cord may predict long-term outcomes in early phases of MS. Advanced MRI techniques, including cortical lesions, chronic active lesions, atrophy and brain microstructural abnormalities can further predict disability progression during the disease course.
• The 2021 MAGNIMS–CMSC–NAIMS international consensus recommendations offer updated guidelines on the utilization of MRI for diagnosing, prognosticating, and monitoring treatment in MS patients.

**Search strategy and selection criteria**

References for this Series paper were identified through searches of PubMed (https://www.ncbi.nlm.nih.gov/pubmed) with the search terms “Automated quantification tolls”, “Artificial intelligence”, “Brain”, “Chronic active lesions”, “Clinically isolated syndrome”, “Cortex”, “Cortical lesions”, “Diagnosis”, “Diagnostic Criteria”, “Differential Diagnosis”, “Grey Matter”, “Guidelines”, “Iron Rim Lesions”, “Lesion/s”, “MRI”, “McDonald criteria”, “Multiple Sclerosis”, “Optic nerve”, “Paramagnetic Rim Lesions”, “Prognosis”, “Primary Progressive”, “Progressive”, “MRI protocol”, “Secondary Progressive”, “Slowly-expanding lesions”, “Spinal Cord”, “Treatment response”, “White Matter”, from 1st January 1979 until 15th January 2024. Only papers published in English were reviewed. The final reference list was generated with the consensus of all co-Authors of this Series article on the basis of originality and relevance to the broad scope of this Series paper, with a focus on articles published during the past five years.

Recently, uncommon groups within the MS population, such as primary progressive (PP) phenotypes, pediatric or late-onset MS, and radiologically isolated syndrome (RIS), have gained increased attention. Furthermore, new potential MRI features for MS diagnosis and prognostication have been proposed, including the assessment of optic nerve involvement, and the detection of cortical lesions (CLs), central vein sign (CVS) and chronic active lesions.

In this Series paper, we outline the current role of MRI in MS diagnosis and prognosis, discussing the potential application of emerging MRI markers and tools.

## Basic principles of MRI techniques

Clinical MRI relies on the resonance signal of hydrogen (^1^H) spins of the intra- and extracellular tissue water. It offers different sequences and techniques to visualize changes in the microstructural environment of these water compartments (Table 1). In MS, most pathophysiological features (i.e., inflammation, edema, demyelination, and axonal loss) are associated with an increase in T_2_ relaxation time. A T_2_-weighted spin echo sequence therefore is the most sensitive approach to depict MS lesions as hyperintense signal intensities.^2^ Improved conspicuity of T_2_ lesions can be achieved with a fluid-attenuated inversion recovery sequence (FLAIR),^2^ especially with three-dimensional (3D) acquisitions.^7^ FLAIR sequences suppress the cerebrospinal fluid (CSF) by an inversion radio frequency pulse followed by a delay time that corresponds to zero-magnetization of the CSF during T_1_ relaxation.

**Table 1:** Conventional and optional sequences for MRI in MS patients.

Contrast	Contrast mechanism	Sequence	Relevance for MS
T_2_-weighted	T_2_ (spin–spin or “transverse”) relaxation	(fast) spin echo or FLAIR, 2D or 3D	Detection of white matter abnormalities
T_1_-weighted	T_1_ (spin-lattice or “longitudinal”) relaxation	2D (fast) spin echo at lower field strength, 3D gradient echo at higher field strengths (≥3 T)	Detection of acute contrast enhancing lesions, assessment of more severe tissue damage (“black holes” and atrophy)
DIR	Two consecutive inversion radiofrequency pulses act as a T_1_-filter	2D or 3D (fast) spin echo	Detection of cortical lesions
Susceptibility-based imaging	Paramagnetic shifts due to iron deposits and deoxygenized blood	T_2_∗-weighted gradient echo sequences, with different acquisition modalities, such as 3D spoiled gradient echo or 3D echo planar imaging (low flip angle, echo time 20–30 ms)	Detection of central vein in MS lesions and iron rims
Magnetization transfer	Proportion of macromolecular bound protons and rate of exchange with tissue water	3D spoiled gradient echo (low flip angle, short echo time) sequence needs to be performed with and without MT saturation pulse	Reduced MT ratio is considered as a marker for demyelination
Diffusion weighted	Restriction of water mobility and orientational effects	Spin echo sequence with diffusion sensitizing gradient and echo planar readout	Sensitive for microstructural damage, higher diffusion models offer more specific assessment

Abbreviations: 2D, two-dimensional; 3D, three-dimensional; DIR, double inversion recovery; FLAIR, fluid-attenuated inversion recovery; MRI, magnetic resonance imaging; MS, multiple sclerosis; MT, magnetization transfer.

T_1_ contrast is considered a more sensitive marker for demyelination and axonal loss since these result in T_1_ prolongation.^8^ T_1_ contrast can be achieved with a short echo time spin echo sequence and a repetition time that corresponds to T_1_ of white matter (WM). However, at higher field strengths, it is advantageous to use gradient echoes with shorter repetition times. This permits true 3D imaging with improved T_1_ contrast and high signal-to-noise ratio. 3D T_1_-weighted sequences with isotropic resolution are also crucial when assessing brain volume and atrophy. T_1_-weighted sequences are still the only way to reliably identify active lesions after gadolinium (Gd)-based contrast administration, which produces a strong paramagnetic moment leading to significant T_1_ shortening. Consequently, active lesions where the blood–brain-barrier becomes leaky appear bright on post-Gd T_1_-weighted images. Whilst inversion recovery (IR) can suppress tissue with a specific T_1_ relaxation time, double inversion recovery (DIR) is used to highlight a specific tissue while suppressing all other magnetization. The DIR sequence provides excellent contrast for the cortex and therefore was introduced to detect cortical MS lesions with improved sensitivity. The DIR sequence is usually performed with fast spin echo readout and provides the best sensitivity when performed in 3D acquisition mode.^9^

Magnetic susceptibility reflects the ability of tissue to become magnetized when placed in magnetic field. Brain tissue is weakly diamagnetic (=non magnetizable) which corresponds to the magnetic properties of water as its main component. In contrast, iron can induce paramagnetic shifts and accentuate MS related features, including lesions with central veins^10^ and iron rims, and iron accumulation in the basal ganglia.^11^ These features can be detected with susceptibility-based imaging, which is performed with T_2_∗-weighted gradient echo sequences, with different possible acquisition modalities, such as 3D spoiled gradient echo or 3D echo planar imaging.^12^ Because susceptibility is encoded within the phase image, susceptibility-based imaging combines magnitude with phase. High spatial resolution and good signal-to-noise ratio are best achieved in 3D acquisition mode and are essential for central vein and iron rim detection. Among susceptibility-based imaging, quantitative susceptibility mapping (QSM) includes imaging techniques that allow to quantify the absolute concentrations of specific elements including iron, calcium, and myelin.^13^ This is achieved by assessing their alterations in local magnetic susceptibility. QSM may offer a better contrast-to-noise ratio for specific tissues and structures when compared to T_2_∗-weighted magnitude images.^13^

Magnetization transfer (MT) imaging and diffusion weighted imaging (DWI) are optional sequences that provide quantitative measures of microstructural tissue changes. MT imaging is based on the magnetization exchange between ^1^H spins bound to macromolecules (including the myelin's proteins and lipids and also other macromolecules) and surrounding tissue water. When using a spoiled gradient echo sequence that is performed with and without an off-resonant radio-frequency pulse that can saturate (null) the longitudinal magnetization of the bound protons, a MT ratio (MTR) can be derived from these two measurements. The MTR is specific for myelin content,^14^ even though it correlates with other pathological substrates, such as axonal density.^15^^,^^16^ The mobility of intra- and extracellular tissue water, driven by Brownian motion, is restricted by cellular structures leading to anisotropic diffusion, particularly in the intracellular cytoplasm of the axons. DWI can pick up alterations in water mobility, and diffusion tensor imaging (DTI) is a fitting model that can assess orientational features such as fiber orientation and the degree of diffusion anisotropy. Complex diffusion models are also able to assess the diffusion properties for intra- and extra cellular water separately.^17^ DWI is based on spin echo sequences with diffusion sensitizing gradients and a segmented or “single shot” echo planar readout.^17^ This type of readout is necessary to limit the impact of physiological and unintentional motion for this highly motion sensitive sequence.

## Diagnostic criteria of MS

**Fig. 1:**
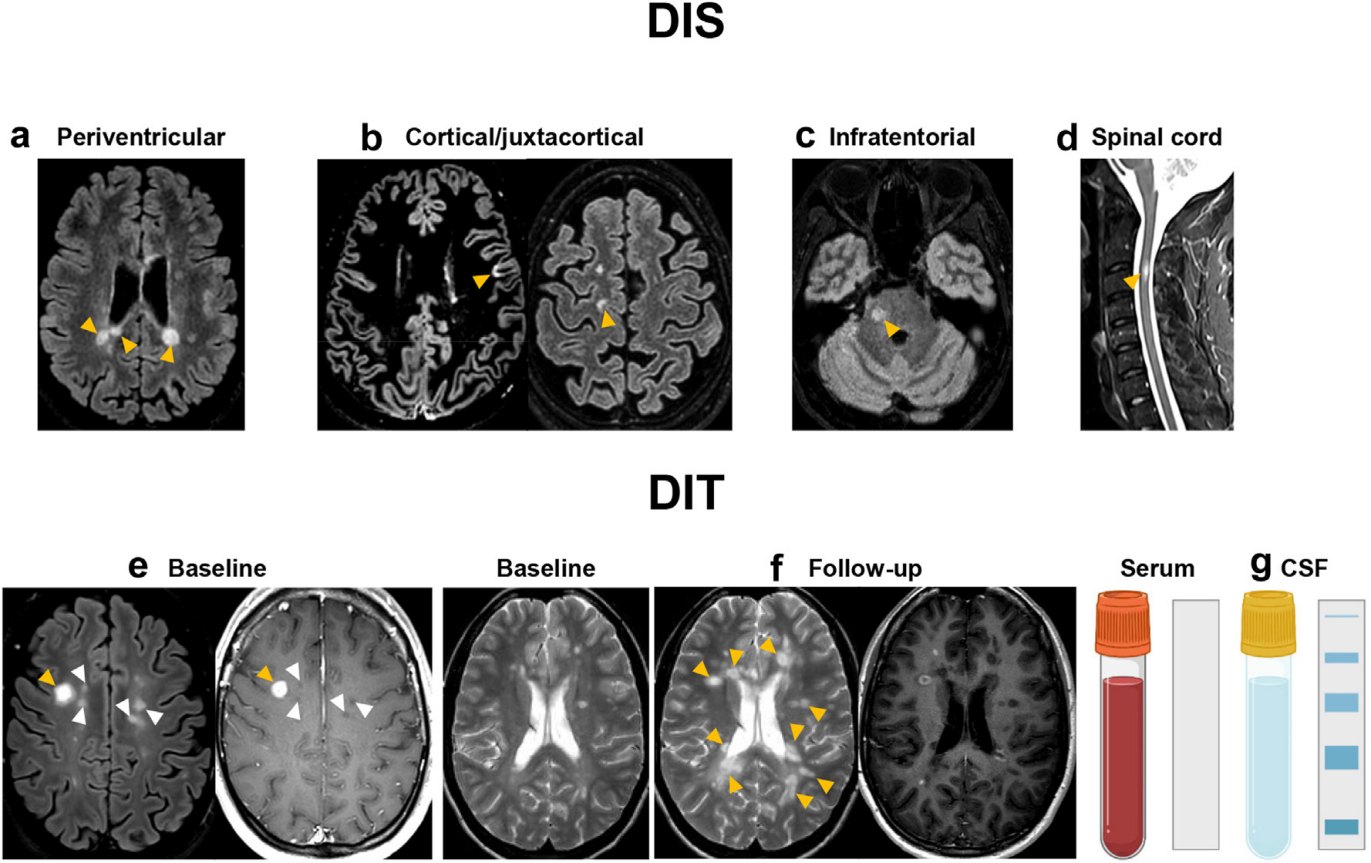
**2017 McDonald criteria for demonstration of DIS and DIT in patients with a CIS suggestive of MS.** Typical MRI examples (orange arrowheads) of (a) periventricular, (b) cortical/juxtacortical, (c) infratentorial and (d) spinal cord MS lesions. DIS can be demonstrated by ≥1 T_2_-hyperintense lesions in ≥2 of 4 typical areas of the central nervous system (i.e., periventricular, cortical/juxtacortical, infratentorial or spinal cord). DIT can be demonstrated by (e) a simultaneous presence of Gd-enhancing (orange arrowhead) and non-enhancing (white arrowheads) lesions at any time; (f) a new T_2_-hyperintense and/or Gd-enhancing lesion on follow-up MRI (orange arrowheads), with reference to a baseline scan, irrespective of the timing of the baseline MRI. For the definition of both DIS and DIT, the distinction between symptomatic and asymptomatic lesions has been removed in the 2017 revision of the McDonald criteria. (g) In patients with a typical CIS suggestive of MS fulfilling DIS criteria and with no better explanation for the clinical presentation, the demonstration of CSF-specific OCBs, i.e., not present in the serum but only in the CSF, substitutes for the requirement of fulfilling DIT, thus allowing a diagnosis of MS, even if the clinical and MRI findings do not meet the criteria for DIT. Abbreviations: CIS, clinically isolated syndrome; CSF, cerebrospinal fluid; DIS, dissemination in space; DIT, dissemination in time; Gd, gadolinium; MRI, magnetic resonance imaging; MS, multiple sclerosis.

In the 2017 revision of the McDonald MRI criteria,^1^ DIS can be demonstrated by ≥1 T_2_-hyperintense lesions in ≥2 of 4 typical areas of the CNS (Table 2, Fig. 1). DIT can be demonstrated by a simultaneous presence of Gd-enhancing and non-enhancing lesions at any time or a new T_2_-hyperintense and/or Gd-enhancing lesion on follow-up MRI (Table 2, Fig. 1).^1^ The modifications introduced in this last revision^1^ included the removal of any distinction between symptomatic and asymptomatic lesions, and the combination of cortical lesions and juxtacortical lesions to expand the concept of juxtacortical involvement. Furthermore, in patients with a typical CIS suggestive of MS fulfilling clinical or radiological DIS, the presence of CSF-specific oligoclonal bands (OCBs) supports DIT (Table 2, Fig. 1).^1^

**Table 2:** The 2017 McDonald criteria for diagnosis of MS.

Clinical presentation	Findings	Additional data needed for MS diagnosis
Relapse-onset (CIS)	≥2 clinical relapses and objective clinical evidence of ≥2 lesions; OR ≥2 clinical relapses and objective clinical evidence of 1 lesion and clear-cut historical evidence of a prior relapse involving a lesion in a distinct anatomic location	None
	≥2 clinical relapses and objective clinical evidence of 1 lesion	DIS, demonstrated by: A second clinical relapse implicating a different CNS site OR demonstration of DIS by MRI (Fig. 1)
	1 clinical relapse and objective clinical evidence of 2 or more lesions	DIT, demonstrated by: A second clinical relapse OR demonstration of DIT by MRI (Fig. 1) Demonstration of CSF-specific OCBs^a^
	1 clinical relapse and objective clinical evidence of 1 lesion	DIS and DIT, demonstrated by: For DIS: A second clinical relapse implicating a different CNS site OR demonstration of DIS by MRI (Fig. 1) For DIT: A second clinical relapse OR Demonstration of DIT by MRI (Fig. 1) Demonstration of CSF-specific OCBs^a^
Progressive-onset (PPMS)	One year of disability progression (retrospectively or prospectively determined) independent of clinical relapse	≥2 out of 3 of the following criteria: • ≥1 T_2_-hyperintense lesions in ≥1 areas in the brain characteristic of MS (periventricular, cortical/juxtacortical or infratentorial) with no distinction between symptomatic or asymptomatic lesions • ≥2 T_2_-hyperintense WM lesions in the spinal cord^,^ with no distinction between symptomatic or asymptomatic lesions • Presence of CSF-specific OCBs

Abbreviations: CIS, clinically isolated syndrome; CNS, central nervous system; DIS, dissemination in space; DIT, dissemination in time; MRI, magnetic resonance imaging; MS, multiple sclerosis; OCB, oligoclonal band; PPMS, primary progressive multiple sclerosis; WM, white matter.

a In patients with a typical CIS suggestive of MS fulfilling DIS criteria and with no better explanation for the clinical presentation, the demonstration of CSF-specific OCBs substitutes for the requirement of fulfilling DIT, thus allowing a diagnosis of MS, even if the clinical and MRI findings do not meet the criteria for DIT.

The 2017 McDonald criteria exhibit higher sensitivity, lower specificity and similar accuracy compared with the previous 2010 revision of the criteria in predicting the second clinical attack (i.e., clinically definite [CD] MS) both in adults and pediatric CIS patients.^18^, ^19^, ^20^, ^21^, ^22^, ^23^ Moreover, the 2017 McDonald criteria substantially shorten time to MS diagnosis, with more CIS patients being diagnosed with MS already at the time of the first clinical manifestation and with a single MRI scan.^18^, ^19^, ^20^^,^^24^ In 785 CIS patients, the 2017 McDonald criteria were found to reduce the median time to diagnose MS by 4.6 years in comparison to the clinical criterion alone (i.e., Poser criteria).^20^ Additionally, they allowed to diagnose MS 10 months before than the 2010 McDonald criteria (3.2 vs 13.0 months).^20^

An early diagnosis allows starting earlier commencement of disease-modifying therapies (DMTs), limiting the accumulation of irreversible clinical disability. In 1174 CIS patients,^25^ the median time from CIS to treatment initiation reduced by 82% from the Poser (i.e., clinical criterion alone)^26^ to the 2017 McDonald criteria^1^ periods and this was associated with a significantly lower risk of reaching an Expanded Disability Status Scale (EDSS) score ≥3.0 for patients diagnosed with the most recent diagnostic criteria.^25^

However, the inappropriate application of MRI diagnostic criteria, especially in the context of atypical clinical presentations, may increase the risk of misdiagnosis.^2^^,^^27^, ^28^, ^29^ To minimize misdiagnosis, standardized MRI protocols,^6^ careful determination of which imaging patterns constitute ‘typical’ or ‘atypical’ MS features and guidelines for a proper interpretation of imaging findings^2^ are crucial.

Careful exclusion of other neurological disorders is essential in the MS diagnostic work-up since the range of diseases mimicking clinical manifestations and MRI features of MS is wide.^2^, ^3^, ^4^ A few MS mimicks, such as neuromyelitis optica spectrum disorder (NMOSD)^30^ and myelin oligodendrocyte glycoprotein-associated disease (MOGAD)^31^ have been more accurately characterized in recent years.^2^, ^3^, ^4^ Moreover, small-vessel disease (SVD), a condition associated with aging and frequently observed in smokers, and in patients with cerebrovascular risk factors (e.g., hypertension, diabetes, dyslipidemia, etc.), may represent the most common differential diagnosis for brain WM hyperintensities especially in older patients who tend to have a greater prevalence of underlying comorbidities.^32^

## Promising MRI measures for MS diagnosis

Recently, more distinctive MRI features have been proposed to improve the specificity and accuracy of MS diagnostic work-up, including CLs, the CVS, and chronic active lesions.

### CLs

**Fig. 2:**
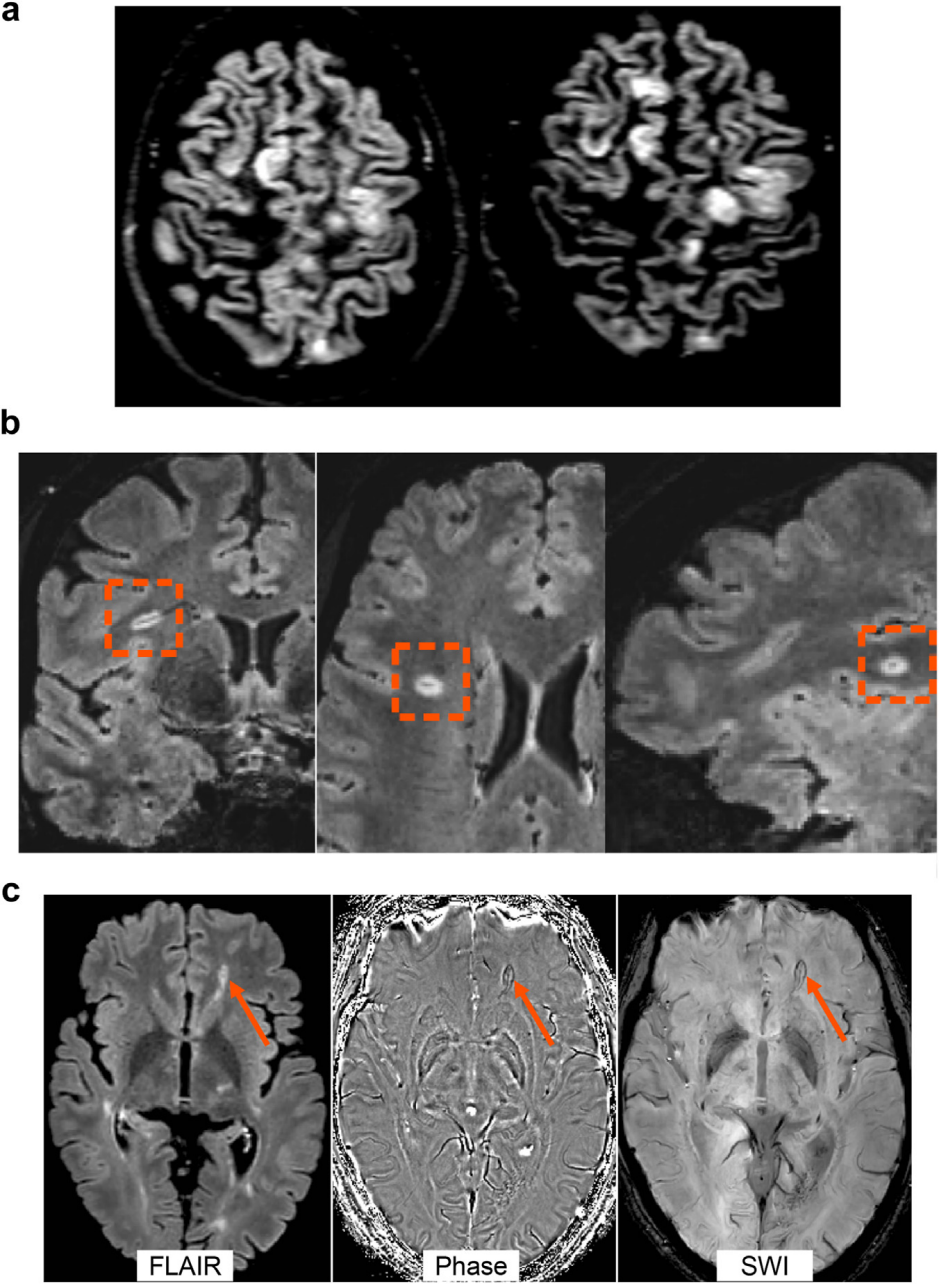
**Examples of cortical lesions, the central vein sign and chronic active lesions**. (a) Several cortical lesions are visible in a multiple sclerosis patient on double inversion recovery sequence at 3.0 T. (b) A white matter lesion showing the central vein sign (orange dotted rectangle) on post-contrast T_2_-FLAIR∗ sequence at 3.0 T. (c) Among the T_2_-hyperintense white matter lesion visible on T_2_-FLAIR, one lesion shows an hypointense rim on phase image and SWI sequence at 3.0 T (orange arrow). Abbreviations: FLAIR, fluid-attenuated inversion recovery; SWI, susceptibility-weighted imaging.

Cortical involvement is an extremely specific hallmark of MS and is highly clinically relevant (Fig. 2a). CLs are present from the early phases of MS, and they accumulate over time.^33^ Several studies confirmed their role in the diagnostic work-up,^34^^,^^35^ especially in differentiating MS from other MS mimics,^2^^,^^36^ and supported their inclusion in the recent revision of the diagnostic criteria.^1^ Imaging of CLs, especially subpial lesions, is technically challenging, mainly because of their characteristics, size and location. Many CLs are still not detected by any MRI technique,^37^ although 7.0 T MRI detects more CLs with respect to the best performing 3.0 T MRI,^38^ and imaging protocols including DIR, phase-sensitive inversion recovery (PSIR), magnetization prepared rapid gradient echo (MPRAGE), magnetization prepared two rapid acquisition gradient echo (MP2RAGE) and fluid and white matter suppression (FLAWS) sequences may substantially improve their *in vivo* detection.^33^^,^^39^^,^^40^

### CVS

Perivenous demyelination is a distinctive pathological feature that is specific of demyelinating disorders of the CNS, especially MS.^41^ The association between brain WM venules and MS lesions (i.e., “CVS”)” can be efficiently visualized in susceptibility-based MRI at varying MRI field strengths and across scanner manufacturers, taking advantage of the T_2_∗-shortening effect of deoxyhemoglobin,^42^ with improved detection after Gd-based contrast (Fig. 2b).^43^^,^^44^ The presence of the CVS may improve the accuracy of MS diagnosis and a threshold of WM lesions showing the CVS between 35% and 50% has been established as the best cutoff to separate MS and non-MS mimics, including SVD, migraine, NMOSD, MOGAD, and other inflammatory CNS disorders.^36^^,^^43^^,^^45^, ^46^, ^47^ To facilitate their assessment, a few studies proposed a threshold of 3 or 6 CVS-positive lesions, which has shown a high sensitivity and good specificity for MS diagnosis.^47^^,^^48^

### Paramagnetic rim lesions

Histopathologically, several MS lesions show a smouldering inflammatory profile which continues after the acute inflammatory phase. Chronic active lesions typically exhibit a combination of ongoing myelin breakdown, remyelination attempts, and infiltrating immune cells, such as lymphocytes and macrophages.^49^ They can be visualized with susceptibility-based imaging as lesions showing an hypointense rim (i.e., paramagnetic rim lesions [PRLs]) since they are usually surrounded by activated microglia, which are rich in iron (Fig. 2c).^46^ PRLs are specific to MS and their assessment can potentially improve the differentiation of MS from other conditions. They have been described in patients with CIS suggestive of MS,^50^ but not in MS mimics, including NMOSD, Susac syndrome, and SVD.^50^, ^51^, ^52^ The presence of at least one PRL was found the optimal cut-off to distinguish CIS/MS patients from MS mimickers and old healthy subjects with a high specificity (99.7%) but low sensitivity (24.0%) and area under the curve [AUC] = 0.71). Of note, the presence of at least one PRL or at least four lesions with CVS improved the sensitivity (57.9%) and AUC (0.83), preserving the specificity (90.6%).^52^ Among CIS patients, fulfilling the “CVS” criteria (≥3 lesions or 40% threshold of lesions with the CVS) and/or having ≥1 chronic active lesions predicted MS conversion within three years with good sensitivity (up to 90.4%) and specificity (up to 85.7%).^50^ Notably, none of the patients who remained with a CIS diagnosis after three years exhibited any chronic active lesion.^50^ QSM has also been applied to identify PRLs. Recent studies showed that QSM may be superior to phase and susceptibility-weighted images in detecting PRLs,^53^ with a prevalence of PRLs ranging from 4.2% to 10.6%.^53^, ^54^, ^55^, ^56^

## Optic nerve involvement: contribution to diagnosis and prognosis

The diagnosis of optic neuritis is typically made after a thorough clinical history and examination. Optic nerve MRI (ON-MRI) can detect T_2_-hyperintense lesions in the optic nerve of MS patients, even in the absence of optic neuritis history. A recent position paper suggests the need to establish the diagnosis of “definite” optic neuritis with paraclinical tests (including ON-MRI among others).^57^ However, while ON-MRI will be of value to rule out other causes of optic neuropathy (e.g., a compressive lesion), the visualization of an optic nerve lesion might not be specific of an inflammatory aetiology of optic neuropathy.^6^^,^^58^, ^59^, ^60^ Thus ON-MRI should be evaluated together with brain and spinal cord MRI findings to help with the differential diagnosis.

**Fig. 3:**
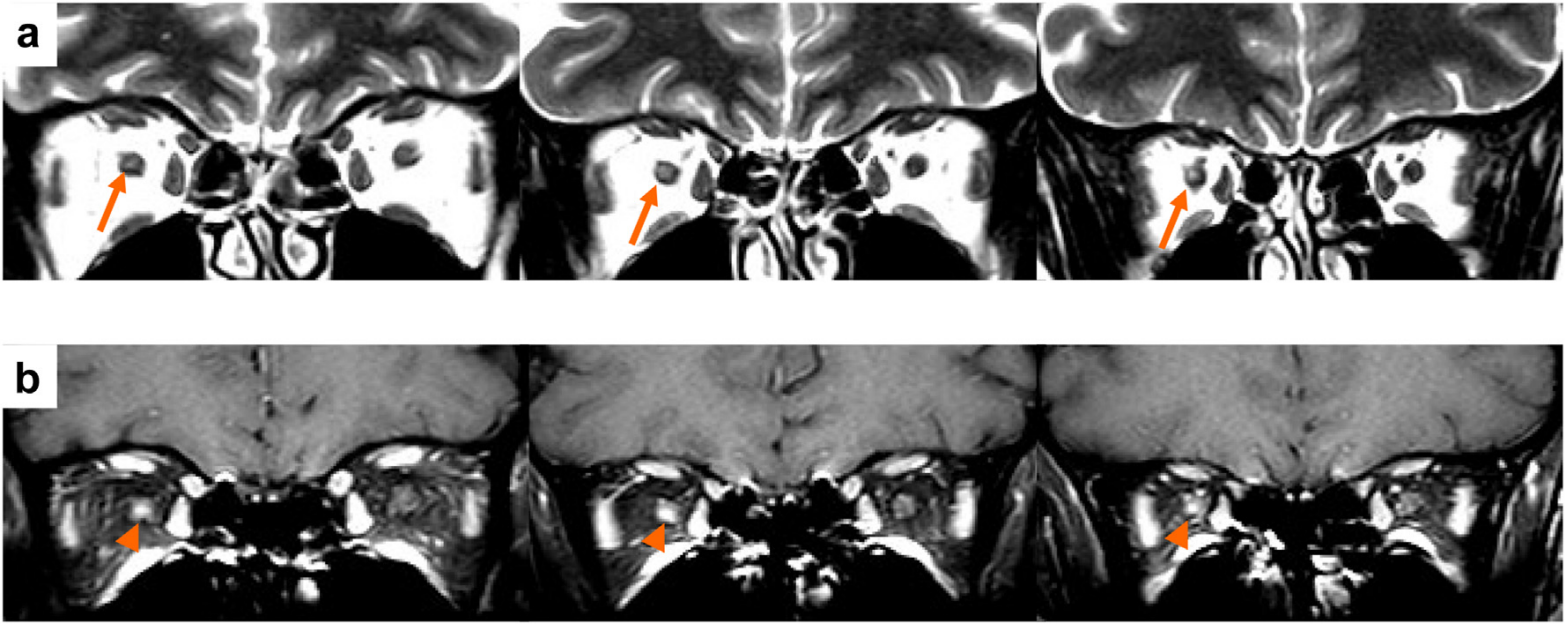
**Example of lesions of the optic nerve in multiple sclerosis**. (a) On coronal fat-suppressed T_2_-weighted sequence, the posterior endo-orbital portion of the right optic nerve is enlarged and shows a hyperintensity (orange arrows), with (b) a focal gadolinium enhancement (orange arrowhead) on post-contrast T_1_-weighted sequence.

The recommended optic nerve protocol includes axial and coronal fat-suppressed T_2_-weighted or short tau inversion recovery (STIR), and fat-suppressed contrast-enhanced T_1_-weighted sequences.^6^ Although higher rates of optic nerve lesion detection have been reported with 3D DIR sequences, especially in asymptomatic eyes,^61^ this superiority has only been evaluated in 3.0 T scans. Studies focusing on acute optic neuritis patients report rates of optic nerve enhancement on contrast-enhanced T_1_-weighted sequences from 34% to 78% (Fig. 3), depending on time elapsed since the onset of optic neuritis, and corticosteroid use. Importantly, in the acute phase, lesion length (but not Gd-enhancement characteristics) was associated with poorer visual recovery 12 months after visual symptom onset.^62^^,^^63^ Moreover, optic nerve lesion length at onset was associated with the degree of retinal damage, as measured by optical coherence tomography (OCT) parameters.^63^

Despite its relevance, the optic nerve has never been considered in the McDonald criteria for MS diagnosis which means that the threshold for MS diagnosis is different for patients presenting with optic neuritis than for patients experiencing other classical CIS symptoms. ON-MRI has been found to detect optic nerve lesions in a high proportion of CIS patients (40.2%–52.3%), especially if they present with an ON (72.7%–100%).^64^^,^^65^ Since the publication of the last revision of the diagnostic criteria,^1^ different studies have evaluated the diagnostic performance of adding the optic nerve as a new region to fulfil DIS criteria.^65^, ^66^, ^67^, ^68^ Optic nerve assessment was defined either by clinical grounds, visual evoked potentials (VEP), OCT, and/or ON-MRI. All these studies demonstrated that fulfilment of modified DIS criteria (including optic nerve information and using a cut-off of 2 out of 5 typical regions) slightly improved the diagnostic performance of current 2017 DIS criteria by increasing sensitivity (especially in patients with optic neuritis) with different degrees of impact on specificity (either no impact or a small drop in specificity that improves when combining modified DIS criteria with DIT).

## Spinal cord involvement: contribution to diagnosis and prognosis

Imaging of the spinal cord in suspected MS patients is important for diagnostic and prognostics purposes as cord lesions are highly specific for MS and finding multiple cord lesions unfavorably affects the disease course. Cord imaging, however, comes with technical challenges due to its small diameter, elongated course and artefacts from CSF pulsation and breathing.^69^ Dedicated surface coils, pre-saturation pulses and dual-contrast pulse-sequences (including proton-density, STIR) can address these challenges.

Short segment cord lesions are typical of MS, thus spinal cord involvement has been included as one of the 4 topographies for assessment of DIS. Spinal cord lesions tend to involve the peripheral WM in the axial plane, though additional grey matter (GM) involvement is not uncommon. However, isolated GM involvement should prompt consideration of NMOSD or MOGAD. New/active lesions may enhance following Gd administration and be associated with some surrounding edema and swelling.

Finding multiple (new) cord lesions not only increases the chances of developing further relapses (and CDMS) but is also an unfavorable prognostic marker and strongly increases the likelihood of developing disability^70^ and secondary progressive (SP) MS.^71^ When there are multiple lesions, they can become confluent and be associated with cord atrophy. Cord atrophy may also develop independent of (visible) cord lesions and especially GM cord atrophy is prognostically unfavorable.^72^

Conversely, there is less evidence that serial cord imaging is helpful to monitor treatment and disease evolution. This is in part due to the technical challenges of cord imaging (which often does not include whole-cord axial slices) making it challenging to detect new lesions with a high degree of certainty. The significant time-investment needed for good-quality cord imaging is offset by the fact that brain imaging is faster and more robust, while being much more likely to reveal new lesions. Accordingly, at present, it is not recommended to routinely perform spinal cord imaging to monitor MS treatment.^6^

## Special situations: PPMS, age extremes and RIS

MS can be diagnosed in certain uncommon situations, with their own challenges, which merit discussion.

### PPMS

About 10–15% of MS cases exhibit gradual clinical progression from onset and are labelled as PPMS. Interestingly, a Swedish cohort study reported a declining rate of PPMS diagnosis as a proportion of MS diagnosis over the decades (from 19% in the 1980s to 2% in the last decade).^73^ PPMS diagnostic confirmation differs slightly from relapsing-remitting (RR) MS with emphasis on the presence of CSF-specific OCBs and ≥2 spinal cord lesions (Table 2).^1^ However, a recent study with 117 PPMS patients showed that sensitivity (89% vs 85%), specificity (100% vs 100%) and accuracy (91% vs 87%) of the 2017 and 2010 McDonald criteria for progressive- and relapse-onset MS were similar, suggesting that a single set of MRI criteria for all MS patients may be applied in the future.^74^

These considerations arose as there is felt to be a greater risk of misdiagnosing PPMS than RRMS, given the relative non-specificity of its clinical onset. PPMS may represent one extreme of a pathophysiological spectrum in MS that is characterized by a more limited blood–brain barrier permeability and acute inflammatory activity and dominated by neurodegenerative processes secondary to a compartmentalized chronic inflammation, mitochondrial dysfunction, and oxidative stress.^75^^,^^76^ Other factors may also influence pathophysiology, e.g., metabolic and microstructural abnormalities without extensive atrophy have been observed in the normal-appearing spinal cord.^77^ The spine is also a sensitive site of pathological change whose atrophy can predict disability progression^78^ and is faster than RRMS.^79^ Other features associated with higher rates of disability progression include higher early GM diffusivity, higher early disability change, new T_1_-hypointense lesions, relapses (although rare), higher age, female sex.^80^^,^^81^ Lesion location at presentation, particularly with spinal cord or brainstem involvement also predicts greater disability progression.^82^ Median time to reach EDSS 6.0 occurs around 8–10 years after onset.^81^^,^^83^ The disability progression rate is similar to SPMS after EDSS 4.0 is reached.^84^

### Pediatric-onset MS (POMS)

POMS (MS onset before 18 years of age) constitutes 3–5% of the MS population^85^^,^^86^ and is rare with an estimated global incidence of 0.87 per 100,000 per year in a recent meta-analysis.^85^ Diagnostic criteria, developed and validated in adults with MS, can be used to diagnose POMS, although caution is advised for acute disseminated encephalomyelitis (ADEM)-like presentations and children under 11 years of age.^1^^,^^86^ This is due to the higher prevalence of MOGAD and monophasic acquired demyelinating syndromes (ADS) in these groups. The 2017 McDonald revision performed very well when applied to a pediatric cohort, with periventricular and T_1_-hypointense lesions helping to distinguish between monophasic ADS and POMS.^21^ POMS exhibits a RR course (98%)^87^ with higher relapse rate and brain lesion accumulation observed early on, than adults with MS.^88^ POMS took longer than adult MS to reach disability milestones (adjusted-hazard ratio [HR] = 0.77) after onset but did so at an earlier age (adjusted-HR = 4–5).^89^ Overall median time to reach EDSS 4.0 is around 30 years after onset and to reach EDSS 6.0 is about 40 years,^89^^,^^90^ but this is influenced by high-efficacy DMTs which delay progression^90^ and show comparable efficacy to adults.^91^

### Late-onset MS (LOMS)

Approximately 3–5% of MS patients present their initial symptoms after the age of 50 years, referred to as LOMS.^92^, ^93^, ^94^ The majority of these patients show a monosymptomatic disease onset, especially with motor or cerebellar impairment.^92^, ^93^, ^94^, ^95^, ^96^, ^97^ Compared to MS patients with a clinical onset at younger age, LOMS is characterized by a lower frequency of clinical relapses and MRI inflammatory activity,^95^ a higher proportion of progressive MS forms (up to 50%), a more severe disease course and a faster disease progression, with a significantly shorter time to reach clinically relevant milestones of disability (EDSS = 3.0 and EDSS = 6.0).^92^, ^93^, ^94^, ^95^, ^96^, ^97^ These unusual clinical features may be due to brain aging effects (e.g., lower neuroplasticity, greater oxidative stress, reduced repair/remyelination, vascular co-morbidities) and immunosenescence phenomena with a lower role for the adaptive relative to the innate immune system in driving MS pathology.^98^

Since MS-related clinical features in LOMS can be potentially confused with symptoms of other diseases more frequently occurring at older age (e.g., SVD), the differential diagnosis in this patient population may be particularly challenging, with high risk of wrong management plans and late treatment.^92^, ^93^, ^94^, ^95^ Accordingly, although the 2017 McDonald criteria can be applied in this population, careful attention to alternative diagnoses and particularly comorbidities is necessary and further studies are needed to validate these criteria in LOMS.^1^

Of note, LOMS is not as responsive to DMTs as adult MS or POMS.^99^ Despite this, DMT withdrawal can still result in faster disability worsening in LOMS, indicating the need for further research in this area.^100^

### RIS

With increasing availability and use of MRI, incidental T_2_-hyperintense WM lesions are increasingly identified on brain and spinal cord imaging.^1^^,^^101^ The term RIS refers to individuals who have no history of clinical symptoms typical of MS but have CNS WM lesions that are highly suggestive of inflammatory demyelination based on their size, number, shape, and location, possibly reflecting individuals with subclinical and prodromal stages of MS.^101^ Incidence of RIS is uncommon (0.8 cases of RIS per 100,000 person-years in Sweden), but approximately 51% of individuals will develop clinical symptoms of MS within 10 years after being deemed RIS, most often with a RR course.^101^

Younger age (<35 years) at the time of RIS identification, male sex, CSF-restricted OCBs or elevated CSF immunoglobulin G (IgG) index, abnormal visual evoked potentials, higher serum neurofilament light chain levels, as well as infratentorial, spinal cord or Gd-enhancing lesions on the index MRI were predictors of a first clinical event at 5 and 10 years, especially in the presence of two or more risk factors,^101^, ^102^, ^103^, ^104^, ^105^ whereas the presence of a higher number of spinal cord lesions is associated with a higher risk of a PPMS course.^104^^,^^106^

Diagnostic criteria for RIS were first proposed in 2009 and were defined by the presence of T_2_-hyperintense, ovoid, 3 mm in length or more, well-defined asymptomatic CNS WM lesions that must fulfill at least three of the four following features: ≥1 Gd-enhancing lesion or ≥9 T_2_-hyperintense lesions; ≥1 infratentorial lesion; ≥1 juxtacortical lesion; and ≥3 periventricular lesions) and should not be related to other diseases.^107^ In 2017, it was suggested that the 2017 McDonald criteria for DIS and DIT in MS could be used in RIS.^1^^,^^108^ More recently, a validation study suggested that when the 2009 RIS criteria are not fulfilled, an individual could be classed as having RIS if they have one or two DIS locations associated with two of the three following features: ≥1 spinal cord lesions, presence of CSF-specific OCBs, or DIT (i.e., new T_2_-hyperintense or Gd-enhancing lesions) on the follow-up MRI.^109^

## Early predictors of long-term outcome

### Conventional MRI measures

**Fig. 4:**
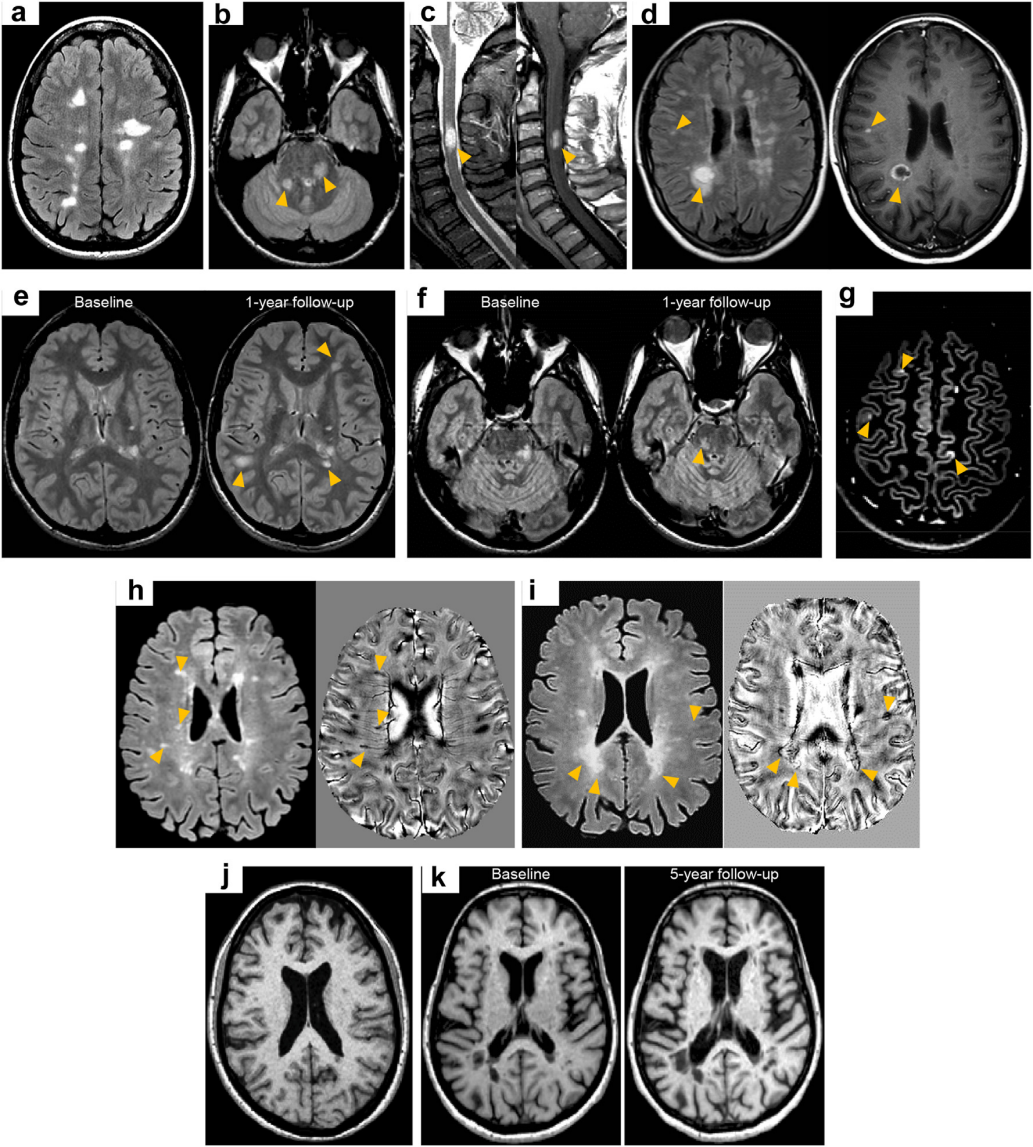
**Summary of early MRI predictors of subsequent worse disease disability progression and evolution to secondary progressive multiple sclerosis at disease onset**. (a) The number and volume of brain T_2_-hyperintense white matter lesions; (b) ≥1 infratentorial lesion (orange arrowheads); (c) ≥1 spinal cord lesion at baseline (orange arrowheads); (d) ≥1 gadolinium-enhancing lesions at baseline (orange arrowheads); (e) increase of T_2_-hyperintense brain white matter lesion number and volume (especially of deep white matter) during the first 1–5 years; (f) ≥1 infratentorial T_2_-hyperintense white matter lesions within 1–3 years (orange arrowheads); (g) ≥1 cortical lesion at baseline (orange arrowheads) on double inversion recovery sequence. Further promising early MRI markers are h) the presence of lesions with the “central vein sign” on susceptibility-based MRI (orange arrowheads); (i) the presence of paramagnetic rim lesions on susceptibility-based MRI (orange arrowheads) (j) presence of substantial brain volume loss at disease onset and/or (k) a faster rate of brain atrophy in the first years of the disease.

Conventional MRI measures including T_2_-hyperintense WM lesion number, location and activity are helpful in establishing long-term prognosis (Fig. 4). A higher number of brain T_2_-hyperientense WM lesions in patients with CIS/early relapsing MS increases the risk of later physical disability and SP disease course, although the relationship is only modest.^110^^,^^111^ Lesion location may offer greater prognostic information. A higher number of infratentorial lesions in patients with CIS/early relapsing MS predicts long-term disability worsening.^112^, ^113^, ^114^ At disease onset, spinal cord MRI can add significant additional prognostic information to brain MRI findings since the evidence of spinal cord lesions has consistently been found to increase the risk of long-term physical disability.^71^^,^^115^^,^^116^ Lesion activity, either the number of Gd-enhancing lesions or short-term changes in T_2_-hyperintense WM lesion number and load during the first 1–5 years of the disease, also predicts long-term physical disability,^71^^,^^110^ and possibly cognitive performance.^71^^,^^117^, ^118^, ^119^ Considering all available conventional MRI measures together may improve patients’ prognosis and treatment decisions. One prospective study found that among CIS patients with no Gd-enhancing lesions and no spinal cord lesions at presentation the estimated risk of SPMS 15 years later was ∼5%, compared with ∼45% in those with at least one spinal cord lesion and ≥2 Gd-enhancing lesions.^71^

Although brain T_2_-hyperintense WM lesion volume progressively increases in untreated MS patients by 5–10% per year,^120^ the correlations between total and new brain T_2_-hyperintense lesions and disability worsening are limited in MS patients with long-standing disease. Limited data regarding the association between new spinal cord lesions and disability progression are also currently available. However, recent studies showed that the presence of new T_2_-hyperintense lesions both in the brain and spinal cord was significantly associated with an increased risk of disability worsening when compared to the absence of new lesions and/or presence of new brain T_2_-hyperintense WM lesions alone (HR = 2.31 and 1.4).^121^^,^^122^

### Advanced MRI techniques

Advanced MRI techniques may further contribute in predicting long-term outcome in the earliest MS phases. CLs have been suggested as one of the primary neuropathological substrates of disease progression, including progression of both cognitive impairment and physical disability.^118^^,^^123^^,^^124^ The number of CLs at disease onset may predict disability progression and conversion to SPMS after 7 years,^123^ as well as cognitive impairment after 20 years.^118^ While no MS patients without CLs at baseline developed SPMS and only a few (1.8%) reached an EDSS score ≥4.0 at last follow-up, the risk of SPMS conversion was progressively higher with an increased number of baseline CLs (HR = 2.16, 4.79, and 12.3 for 2, 5, and 7 CLs, respectively), and time to progression was earlier (median time = 6.5 years in MS patients with ≥7 CLs, on average 4 years earlier than those with 1–3 CLs).^123^ Moreover, the presence of at least 3 CLs at baseline was associated with a higher risk of cognitive impairment at 20 years (odds ratio = 3.7, AUC = 0.67, specificity = 75%, sensitivity = 55%).^118^ On the other hand, after 15 years of the disease, patients who still demonstrated a moderate disability without cognitive impairment showed a remarkably lower CL number and volume increase compared to early RRMS.^123^^,^^125^ CLs have a prognostic role also in MS patients with longer disease duration since their number and volume predict disease worsening^126^ and their accumulation are associated with disability progression in both relapse-onset and PPMS patients.^126^, ^127^, ^128^, ^129^

PRLs are not only relevant during the diagnostic process but also serve as markers of smouldering activity being associated with more severe clinical disability, progression of brain atrophy and disability.^51^^,^^130^ MS patients with ≥4 PRLs had more severe disability, a faster disease progression, with an earlier development of motor and cognitive impairment, and a more severe brain volume loss, thus suggesting a more aggressive disease course.^51^^,^^130^ Moroever, PRL volume was one of the most relevant predictors of EDSS worsening after 3 years.^131^ Accordingly, monitoring these lesions over time can provide valuable information on disease progression, with the potential to guide treatment decisions and help assess response to treatment.^130^, ^131^, ^132^

Chronic active lesions slowly increase in size over time (i.e., slowly-expanding lesions [SEL]), thus they have been identified among those WM lesions showing a linear and progressive expansion over long-enough periods of time on conventional T_1_-and T_2_-weighted sequences.^75^^,^^133^, ^134^, ^135^ The prevalence of SELs seems similar in PPMS (69.0%–73.2%)^75^^,^^133^ and relapse-onset MS patients (59.6%–98.6%).^133^^,^^136^, ^137^, ^138^ Microstructural tissue damage, as quantified by lower MTR, lower normalized T_1_ intensity and higher radial diffusivity, is greater in SELs than in T_2_-hyperintense lesions not expanding over time.^75^^,^^133^^,^^135^^,^^136^^,^^138^ Moreover, SEL number and volume and their intrinsic microstructural tissue abnormalities have been shown to predict disability progression in relapse-onset MS and PPMS.^75^^,^^134^^,^^136^^,^^137^^,^^139^ However, at present, the relevance of SELs in the diagnostic work-up of MS as well as their prognostic role at disease onset have not been explored yet.

Interestingly, SELs and PRLs overlap, but only partially, suggesting that these lesions may represent distinct stages of MS pathology within chronic active lesions.^140^

More severe brain microstructural abnormalities and volume loss at disease onset and a faster rate of brain atrophy in the first years of the disease may also predict long-term MS evolution and cognitive decline in relapse-onset MS. Change within the first 5 years of disease in medullary width significantly predicted disability worsening, SPMS conversion or MS-related death after 30 years, whereas third ventricular width significantly predicted disability progression.^141^ Baseline normal-appearing brain tissue MTR and brain parenchymal fraction and 2-year change in ventricular fraction significantly predicted changes in memory functions after 7 years,^117^ whereas a faster rate of brain atrophy in the first year predicted cognitive performance after 5 years.^119^ Brain and spinal cord volume and atrophy are also useful prognostic markers in more advanced phases of the disease.^142^ In particular, cerebral GM volume loss is an important predictor of long-term prognosis.^142^ In relapse-onset MS, baseline GM volume predicted EDSS worsening after 13 years,^143^ whereas a model including GM mean diffusivity and GM atrophy predicted 15-year disability in PPMS patients.^80^ Spinal cord volume loss progresses at a faster rate in MS patients experiencing disability worsening than those who do not,^142^ and it predicts disability worsening.^70^

## MRI to select treatment and predict treatment response

Treatment selection for each MS patient is typically influenced by many factors, including demographic features (i.e., sex and age), clinical characteristics (severity of clinical relapses, higher relapse rate, brainstem or spinal cord onset, higher disability or a progressive disease course, comorbidities, etc.), and the presence of negative MRI prognostic factors that have been previously discussed.^144^ Accordingly, high efficacy disease-modifying therapies may represent the appropriate therapeutic approach in the presence from the earliest phases of MS of negative prognostic factors associated with long-term disease progression.^144^^,^^145^

MRI represents also a fundamental paraclinical tool to monitor and predict treatment response. MRI is highly sensitive to occurrence of Gd-enhancing lesions or new T_2_-hyperintense lesions forming over time that represent a valid surrogate marker of clinical relapses.^146^ Moreover, MRI can quantify the amount of neurodegeneration (demonstrated by atrophy of the brain and spinal cord) that represents a surrogate marker of irreversible disability progression.^147^

The evidence of ongoing inflammatory activity detected with MRI may predict subsequent disease evolution. Post-hoc analyses of randomized clinical trials and observational studies have shown that the occurrence of MRI activity in the first 6–12 months after treatment start is associated with higher risks of clinical relapses or disability worsening over the short or medium-term period, thus suggesting for a possible treatment change.^148^ Recently, composite outcome measures have been proposed to incorporate both clinical (relapses and disability worsening) and MRI measures (new T_2_-hyperintense and Gd-enhancing lesions) to better predict treatment response and disease evolution.^148^^,^^149^ These include the Rio score,^150^ the modified Rio score,^151^ the MAGNIMS score,^152^ and no evidence of disease activity 3^153^ (NEDA-3). A subsequent iteration of NEDA-3 (i.e., NEDA-4) requires also the absence of brain atrophy, defined according to an annual brain volume loss threshold of 0.4%.^154^ Thanks to the combination of clinically-relevant markers, these composite scores may be more stringent regarding acceptable levels of disease activity while on treatment and may predict medium- and long-term disease evolution with better accuracy compared to single clinical and MRI criteria.^148^ However, since they have been evaluated with specific drugs, their generalizability to other treatments still need to be explored.^148^

## MRI protocol and report in MS diagnosis

The 2021 MAGNIMS–CMSC–NAIMS international consensus recommendations offer revised guidelines on the use of MRI in MS patients that encompass the appropriate methods and timing for employing MRI in the diagnosis of MS, placing particular emphasis on implementing standardized MRI protocols and reports (Table 3, Panel 1).^6^

**Table 3:** Recommended standardized brain, optic nerve, and spinal MRI protocols for MS diagnosis.

	Brain	Spinal cord	Optic nerve
**Suggested MRI parameters**			
Field strength	≥1.5 T (preferably 3.0 T)	≥1.5 T (3.0 T has no added value compared with 1.5 T)	≥1.5 T
Slice thickness	For 3D imaging: 1 mm isotropic is preferred but, if over contiguous (through plane and in plane), not >1.5 mm, with 0.75 mm overlap For 2D imaging: ≤3 mm with no gap (except for diffusion-weighted imaging, for which the slice thickness should be ≤ 5 mm with a 10–30% gap)	Sagittal slices should be ≤ 3 mm with no gap; axial slices should be ≤ 5 mm with no gap	≤2–3 mm with no gap
In-plane resolution	≤1 mm × 1 mm	≤1 mm × 1 mm	≤1 mm × 1 mm
Coverage	Whole brain (include as much of cervical cord as possible)	Cervical and thoraco-lumbar spinal cord, to include conus	Optic nerve and optic chiasm
Axial scan orientation	Subcallosal plane to prescribe (i.e., for 2D imaging) or reformat (i.e., for 3D imaging) axial oblique slices	Perpendicular to the sagittal axis of the spinal cord	Aligned to the orientation of the optic nerve and optic chiasm
**MRI sequence**			
Recommended	Axial T_2_-weighted (TSE or FSE) sequences^a^	At least two of: sagittal T_2_-weighted sequences (TSE or FSE), PD-weighted sequences (TSE or FSE), or STIR	
	Sagittal T_2_-weighted FLAIR (preferably 3D; fat suppression is optional)		
	Axial T_2_-weighted FLAIR (unnecessary if a sagittal 3D FLAIR with multiplanar reconstruction is obtained; fat suppression is optional)	Sagittal T_1_-weighted sequences (TSE or FSE) after contrast^b^	
	Axial (or 3D sagittal) T_1_-weighted sequences after contrast^b^		
Optional	Diffusion-weighted imaging	Sagittal 3D heavily T_1_-weighted sequences (PSIR or magnetisation-prepared rapid acquisition of gradient echoes^c^) only for the cervical segment	Axial and coronal fat-suppressed T_2_-weighted sequences or STIR of optic nerve^d^
	DIR or PSIR for detecting cortical or juxtacortical lesions	Axial T_2_-weighted (TSE or FSE) or gradient-recalled echo^e^	
	High-resolution T_1_-weighted sequences (isotropic 3D acquisition; for quantitative assessment of brain volume)	Sagittal T_1_-weighted sequences (TSE or FSE) before contrast	Axial and coronal fat-suppressed T_1_-weighted sequences post contrast of optic nerve^d^
		Axial T_1_-weighted sequences (TSE or FSE) after contrast^b^	

Abbreviations: 2D, two-dimensional; 3D, three-dimensional; DIR, double inversion recovery; FLAIR, fluid-attenuated inversion recovery; FSE, fast spin echo; PD, proton density; PSIR, phase-sensitive inversion recovery; TSE, turbo spin echo; STIR, short tau inversion recovery.

a A dual echo (proton density-weighted and T_2_-weighted) sequence can be considered as an alternative to a single echo T_2_-weighted sequence.

b Standard doses of 0.1 mmol/kg bodyweight, macrocyclic gadolinium chelates only, with a minimum delay of 5–10 min.

c One of these sequences could replace T_2_-weighted sequences, proton density-weighted sequences, or short tau inversion recovery.

d The acquisition of this sequence can be considered in some clinical situations; 2D or 3D acquisition.

e To corroborate, characterize, and confirm lesions detected on sagittal images or to detect lesions in spinal cord segments with high clinical suspicions of involvement.^6^

*Panel 1* **Recommendations for MRI application in the MS diagnostic work-up.**

**Standardized brain MRI protocol**

• At least 1.5 T; 3.0 T if available.
• Core sequences are: T2-weighted 3D-FLAIR, axial T2-weighted, and T1-weighted with Gd (Table 3).
• Pre-contrast T1-weighted sequences are not required.

**Recommended use of brain MRI sequences**

• To establish the diagnosis according to 2017 McDonald criteria: detection of symptomatic or asymptomatic brain lesions in typical CNS regions to show DIS and DIT.
• To predict future disease activity and disease progression.

**Standardized spinal cord MRI protocol**

• 1.5 T or 3.0 T.
• Details on pulse sequences can be found in Table 3.

**Recommended use of spinal cord MRI sequences**

• To establish MS diagnosis according to 2017 McDonald criteria
  o *Relapse-onset (CIS)*
    ▪ Detection of symptomatic or asymptomatic spinal cord lesions to show DIS and DIT.
    ▪ Differential diagnosis in case of inconclusive brain MRI findings: presence of typical demyelinating spinal cord lesions and exclusion of alternative diagnosis (e.g., NMOSD and MOGAD).
  o *Progressive-onset (PPMS)*
    ▪ Detection of typical demyelinating spinal cord lesions to show DIT.
    ▪ Detection of diffuse lesions.
    ▪ Exclusion of alternative diagnosis.
• To predict future disease activity and disease progression.

**Recommended use of Gd-based contrast agents**

• To show dissemination in time on the baseline MRI scan.
• To contribute to differential diagnosis (i.e., on the basis of the pattern of enhancement).
• To predict future disease activity and disease progression.
• For phenotyping patients with progressive disease (i.e., active or inactive), if a recent (i.e., within 1 year) MRI is not available, and if this information affects treatment decisions.

**MRI in pediatric MS population**

• The same standardized brain and spinal cord MRI protocols as for adults should be acquired.
• Gd-enhanced images are valuable to exclude non-MS diagnosis at onset.
• Full spinal cord MRI should be acquired for diagnosis of children with spinal cord symptoms or signs or with inconclusive brain MRI findings.
• Spinal cord MRI could be obtained to provide a baseline MRI for all pediatric MS patients.
• Dedicated optic nerve MRI is not recommended, except for differential diagnosis with MOGAD or NMOSD and if clinical features are atypical.

**Additional or advanced MRI**

• Diffusion-weighted imaging cannot replace Gd as a marker for active inflammation.
• Dedicated optic nerve MRI is not recommended except for differential diagnosis with NMOSD, MOGAD and in patients with atypical clinical features.
• There is insufficient current evidence to recommend routine use of quantitative MRI techniques and brain volumetric measurements, DIR or PSIR for CLs, and CVS and chronic active lesions as diagnostic markers.

**Image interpretation and report**

• Standardized image interpretation and reporting is recommended.
• Knowledge about definition of lesion types is crucial and warning signs against a diagnosis of MS should be recognized.
• Standard measures, such as T_2_-hyperintense WM lesion count, size and topography and Gd-enhancing lesion count if Gd was administered, are recommended.

Separate identification of cortical lesions (together with juxtacortical lesions) based on standard images (e.g., FLAIR; DIR or PSIR sequences are optional) is recommended.

Abbreviations: CIS, clinically isolated syndrome; CL, cortical lesion; CNS, central nervous system; CVS, central vein sign; DIR, double inversion recovery; DIS, dissemination in space; DIT, dissemination in time; FLAIR, fluid-attenuated inversion recovery; Gd, gadolinium; MOGAD, myelin oligodendrocyte glycoprotein-associated disease; MRI, magnetic resonance imaging; MS, multiple sclerosis; NMOSD, neuromyelitis optica spectrum disorder; PP, primary progressive; PSIR, phase-sensitive inversion recovery; WM, white matter.^6^

Even though 3.0 T MRI scanners are preferred since they improve brain lesion detection and may reduce acquisition times, those with 1.5 T are still sufficient for lesion detection, given good quality scans with proper signal-to-noise ratio and high spatial resolution (Table 3, Panel 1). For diagnosis, recommended brain sequences include T_2_-weighted 3D-FLAIR, axial T_2_-weighted, and post-Gd T_1_-weighted (Table 3, Panel 1). In particular, sagittal T_2_-weighted 3D-FLAIR sequence is considered to be the core sequence for MS diagnosis due to its high sensitivity to WM lesions. 3D acquisitions are preferred to two-dimensional (2D) acquisition since they improve lesion detection and allow the reconstruction on different planes. In case of unavailability of 3D T_2_-weighted FLAIR sequence, high quality 2D pulse-sequences can be acceptable alternatives. Although some safety concerns were recently raised regarding the use of Gd-based contrast agents, post-contrast sequences are recommended in the diagnostic work-up of MS to show DIT and to exclude alternative diagnoses. To detect enhancing lesions, the recommended dose of Gd-based contrast agents is 0.1 mmol/kg body weight and the time delay between contrast administration and T_1_-weighted acquisition should not be shorter than 5 min.

Unlike brain MRI, there is no evidence that using higher field strengths (e.g., 3.0 T) results in a greater detection of spinal cord lesions than lower field strengths. The standardized spinal cord MRI protocol must include at least two of the following three sagittal sequences: T_2_-weighted, proton density-weighted, or STIR (Table 3, Panel 1). A combined acquisition increases the sensitivity of lesion identification but also limits the risk of artefacts and false positive findings. Sagittal MRI scans should ideally cover the whole spinal cord since lesions can be detected in all cord levels, including the conus. Even though Gd-enhancing spinal cord lesions are less common than in the brain, sagittal Gd-enhanced T_1_-weighted spin echo sequence should be also acquired in the diagnostic work-up of MS. Although axial images of the spinal cord are optional, axial T_2_-weighted spin echo sequences can further improve diagnostic certainty, ameliorating the accuracy of lesion detection and differentiating MS from mimics (e.g., NMOSD, MOGAD) on the basis of lesion extension and topography.

Additional brain MRI sequences, including DWI, pre-contrast 3D T_1_-weighted for volumetric measurements, DIR or PSIR to identify CLs, and susceptibility-based imaging to identify CVS and chronic active lesions are currently optional. Similarly, dedicated optic nerve MRI is not recommended except for differential diagnosis with NMOSD, MOGAD and in patients with atypical clinical features (Table 3, Panel 1).

The interpretation of MRI findings should be performed by (neuro)radiologists, with expertise in the identification of neuroimaging features typical and atypical for MS^2^ (Panel 1). Moreover, a standardized radiological report is recommended. It should accurately report standard measures, such as lesion count, size and, topography of T_2_-hyperitense WM lesions, Gd-enhancing lesion count, fulfillment of diagnostic criteria as well as possible incidental findings (Panel 1).^6^

## Novel technologies: contribution to diagnosis and prognosis

### Artificial intelligence

Recent improvements in technologies and the availability of large amount of data have promoted the application of artificial intelligence (AI) algorithms for the identification and segmentation of lesions, the diagnostic-work up of MS, and the characterization of disease course and prognosis.^155^

Numerous segmentation methods employing various approaches have been proposed for quantifying WM lesions.^155^, ^156^, ^157^, ^158^ Among them, image segmentation using deep learning (DL) approaches (typically convolutional neural networks [CNNs]) have been extensively used.^156^ These methods have been demonstrated to be accurate, allowing also to obtain lesion volumes in a more reproducible way and with more limited time-consuming compared to manual quantifications.

Recent application of AI for MRI analysis in MS included also the automated assessment of specific lesion types, such as cortical lesions,^159^ enhancing lesions,^160^ as well as of lesional features, including the presence of the CVS in WM lesions,^161^^,^^162^ and PRLs.^163^^,^^164^

Using DL or machine learning (ML) approaches, recent studies were able to discriminate between MS patients and HC with an accuracy between 70.2% and 98.8% from structural or resting state functional MRI sequences.^165^, ^166^, ^167^, ^168^, ^169^, ^170^, ^171^

Algorithms based on AI may also play a crucial role in distinguishing MS from other conditions. A random forest algorithm, using brain GM imaging measures, achieved 74% accuracy in discriminating NMOSD from MS.^172^ Multiparametric approaches, incorporating data from FLAIR, brain GM, DTI, resting state functional MRI plus clinical and neuropsychological information improved up to 88%.^172^^,^^173^ ML and DL algorithms applied to brain FLAIR and T1-weighted sequences and clinical information showed high accuracy in differentiating MS from NMOSD, migraine, CNS vasculitis, and non-inflammatory WM disorders.^174^, ^175^, ^176^, ^177^

ML algorithms also demonstrated high accuracy in distinguishing MS from non-inflammatory disorders and predicting conversion from CIS to clinically definite MS with an accuracy between 67.6% and 92.9%.^178^, ^179^, ^180^, ^181^

By integrating clinical and MRI data, several studies demonstrated that ML and DL algorithms can accurately predict disability worsening in the different clinical phenotypes and after variable follow-up durations, ranging from six months to fifteen years.^80^^,^^143^^,^^182^, ^183^, ^184^, ^185^ Notably, while lesion measures appear crucial for short-term prediction of disease progression, at long-term follow-up, GM damage becomes a more substantial and significant contributor.

### Automated quantification tools

Manual quantification of brain lesions and volumes is a time-consuming process that is susceptible to errors and high variability. The availability of commercially accessible automated quantification and reporting tools has grown in recent years.^186^ They have the potential to improve the sensitivity, precision and consistency of MRI analysis, possibly reducing time for data analysis. These automatic tools may potentially facilitate the cross-sectional and longitudinal evaluation of volumetric data of each patient against a reference population. This may be beneficial for clinicians in various aspects such as diagnostic work-up, predicting disease progression, monitoring disease evolution and treatment responses. Numerous automated quantification and reporting tools designed for MS have been created for clinical use, and many of these tools have received regulatory approval (see^186^ for a comprehensive and up-to-date review). However, to broaden their application in the clinical setting, standardization and validation using diverse input data remain essential. Moreover, these tools should be intended to provide support and should not replace the direct evaluation performed by clinicians.

## Conclusions

Accurate criteria in the diagnostic work-up of MS are pivotal to facilitate early diagnosis and minimize the risk of misdiagnosis. The 2017 McDonald criteria exhibit high sensitivity and accuracy in predicting the occurrence of a second clinical attack (i.e., clinically-definite MS). They enable timelier MS diagnosis and treatment, but they should be applied only after alternative diagnoses have been carefully ruled out.

To enhance the diagnostic process further, emerging imaging markers (e.g., optic nerve involvement, CLs, CVS, and chronic active lesions) have been proposed. These aim to augment the specificity and accuracy of MS diagnostic criteria. However, before their incorporation into clinical practice, rigorous validation and standardization are still necessary. Moreover, there is a relative lack in knowledge for PPMS, POMS and LOMS which represent rarer presentations. Future research focusing on these areas in large or multi-center settings is necessary.

Several MRI markers, encompassing the number, size, and distribution of focal WM lesions, along with more specific advanced MRI features, serve as early and reliable predictors of subsequent disease progression. Thus, their thorough assessment can potentially identify MS patients at elevated risk of disease progression, necessitating prompt use of highly effective therapies.

To ensure consistency and improvement in MRI use in the diagnostic work-up of MS, it is essential to implement standardized brain and spinal cord MRI protocols. Additionally, accurate interpretation by specialized (neuro)radiologists with expertise in MS neuroimaging features is crucial for achieving a consistent and accurate use of MRI in the context of MS.

## Contributors

Concept and design of the Series paper: M.A. Rocca, M. Di Filippo.

Interpretation of data and drafting of the Series paper: M.A. Rocca, P. Preziosa, W. Brownlee, F. Barkhof, M. Calabrese, N. De Stefano, C. Granziera, S. Ropele, A.T. Toosy, À.Vidal-Jordana, M. Filippi, M. Di Filippo.

Critical revision of the manuscript for important intellectual content: M.A. Rocca, P. Preziosa, W. Brownlee, F. Barkhof, M. Calabrese, N. De Stefano, C. Granziera, S. Ropele, A.T. Toosy, À.Vidal-Jordana, M. Filippi, M. Di Filippo.

## Declaration of interests

Maria A. Rocca received consulting fees from Biogen, Bristol Myers Squibb, Eli Lilly, Janssen, Roche; and speaker honoraria from AstraZaneca, Biogen, Bristol Myers Squibb, Bromatech, Celgene, Genzyme, Horizon Therapeutics Italy, Merck Serono SpA, Novartis, Roche, Sanofi and Teva. She receives research support from the MS Society of Canada, the Italian Ministry of Health, the Italian Ministry of University and Research, and Fondazione Italiana Sclerosi Multipla. She is Associate Editor for *Multiple Sclerosis and Related Disorders*.

Paolo Preziosa received speaker honoraria from Roche, Biogen, Novartis, Merck Serono, Bristol Myers Squibb, Genzyme, Horizon and Sanofi. He has received research support from Italian Ministry of Health and Fondazione Italiana Sclerosi Multipla.

Frederik Barkhof acts in Steering committee or Data Safety Monitoring Board member for Biogen, Merck, ATRI/ACTC and Prothena. Consultant for Roche, Celltrion, Rewind Therapeutics, Merck, IXICO, Jansen, Combinostics. Research agreements with Merck, Biogen, GE Healthcare, Roche. Co-founder and shareholder of Queen Square Analytics LTD. FB is supported by the NIHR Biomedical Research Centre at UCLH.

Wallace Brownlee has received speaker honoraria and/or acted as a consultant for Biogen, Merck, Novartis, Roche, Sandoz, Sanofi and Viatris. WB is supported by the NIHR University College London Hospitals Biomedical Research Centre.

Massimiliano Calabrese received speaker honoraria and travel grants from Biogen, Bristol Myers Squibb- Celgene, Sanofi-Genzyme, Merck Serono, Novartis Pharma, and Roche and received research support from the Progressive MS Alliance and Italian Minister of Health and from Biogen, Bristol Myers Squibb-Celgene, Novartis Pharma, Sanofi-Genzyme, Merck Serono, and Roche.

Nicola De Stefano declared no competing interests.

Cristina Granziera's employer (University Hospital Basel) has received the following fees which were used exclusively for research support: advisory boards and consultancy fees from Actelion, Novartis, Genzyme-Sanofi, GeNeuro, Hoffmann La Roche, Merck and Siemens Healthineers; speaker fees from Biogen, Hoffmann La Roche, Teva, Novartis, Janssen, Merck and Genzyme-Sanofi; and research grants from Hoffmann La Roche, GeNeuro, Genzyme, Novartis and Biogen. CG is supported by the Swiss National Fund n. PP00P3_206151, the Hasler Foundation and the Stiftung zur Förderung der gastroenterologischen und allgemeinen klinischen Forschung.

Stefan Ropele declared no competing interests.

Ahmed T Toosy reports receiving speaker honoraria from Merck, Bayer, Biomedia, and Serono Symposia International Foundation; receiving meeting expenses from Merck, Biogen Idec; and being the United Kingdom principal investigator for two clinical trials sponsored by MedDay Pharma. ATT is supported by recent grants from the MRC (MR/S026088/1), NIHR BRC (541/CAP/OC/818837) and RoseTrees Trust (A1332 and PGL21/10079).

Àngela Vidal-Jordana has received support has received support for contracts Juan Rodes (JR16/00024) and from Fondo de Investigación en Salud (PI17/02162 and PI22/01589) from Instituto de Salud Carlos III, Spain, and has engaged in consulting and/or participated as speaker in events organized by Roche, Novartis, and Merck.

Massimo Filippi is Editor-in-Chief of the *Journal of Neurology*, Associate Editor of *Human Brain Mapping*, *Neurological Sciences,* and *Radiology*; received compensation for consulting services from Alexion, Almirall, Biogen, Merck, Novartis, Roche, Sanofi; speaking activities from Bayer, Biogen, Celgene, Chiesi Italia SpA, Eli Lilly, Genzyme, Janssen, Merck-Serono, Neopharmed Gentili, Novartis, Novo Nordisk, Roche, Sanofi, Takeda, and TEVA; participation in Advisory Boards for Alexion, Biogen, Bristol-Myers Squibb, Merck, Novartis, Roche, Sanofi, Sanofi-Aventis, Sanofi-Genzyme, Takeda; scientific direction of educational events for Biogen, Merck, Roche, Celgene, Bristol-Myers Squibb, Lilly, Novartis, Sanofi-Genzyme; he receives research support from Biogen Idec, Merck-Serono, Novartis, Roche, the Italian Ministry of Health, the Italian Ministry of University and Research, and Fondazione Italiana Sclerosi Multipla.

Massimiliano Di Filippo participated on advisory boards and steering committees for and received speaker or writing honoraria, research support and funding for travelling from Alexion, BMS, Bayer, Biogen Idec, Genzyme, Horizon, Merck, Mylan, Novartis, Roche, Siemens Healthineers, Teva and Viatris.

Role of the funding source: None.

## References

[1] Thompson A.J., Banwell B.L., Barkhof F. (2018). Diagnosis of multiple sclerosis: 2017 revisions of the McDonald criteria. Lancet Neurol.

[2] Filippi M., Preziosa P., Banwell B.L. (2019). Assessment of lesions on magnetic resonance imaging in multiple sclerosis: practical guidelines. Brain.

[3] Geraldes R., Ciccarelli O., Barkhof F. (2018). The current role of MRI in differentiating multiple sclerosis from its imaging mimics. Nat Rev Neurol.

[4] Solomon A.J., Arrambide G., Brownlee W.J. (2023). Differential diagnosis of suspected multiple sclerosis: an updated consensus approach. Lancet Neurol.

[5] McDonald W.I., Compston A., Edan G. (2001). Recommended diagnostic criteria for multiple sclerosis: guidelines from the international panel on the diagnosis of multiple sclerosis. Ann Neurol.

[6] Wattjes M.P., Ciccarelli O., Reich D.S. (2021). 2021 MAGNIMS-CMSC-NAIMS consensus recommendations on the use of MRI in patients with multiple sclerosis. Lancet Neurol.

[7] Patzig M., Burke M., Bruckmann H., Fesl G. (2014). Comparison of 3D cube FLAIR with 2D FLAIR for multiple sclerosis imaging at 3 Tesla. Röfo.

[8] van Walderveen M.A., Kamphorst W., Scheltens P. (1998). Histopathologic correlate of hypointense lesions on T1-weighted spin-echo MRI in multiple sclerosis. Neurology.

[9] Geurts J.J., Pouwels P.J., Uitdehaag B.M., Polman C.H., Barkhof F., Castelijns J.A. (2005). Intracortical lesions in multiple sclerosis: improved detection with 3D double inversion-recovery MR imaging. Radiology.

[10] Sati P., George I.C., Shea C.D., Gaitan M.I., Reich D.S. (2012). FLAIR∗: a combined MR contrast technique for visualizing white matter lesions and parenchymal veins. Radiology.

[11] Langkammer C., Liu T., Khalil M. (2013). Quantitative susceptibility mapping in multiple sclerosis. Radiology.

[12] Mittal S., Wu Z., Neelavalli J., Haacke E.M. (2009). Susceptibility-weighted imaging: technical aspects and clinical applications, part 2. AJNR Am J Neuroradiol.

[13] Granziera C., Wuerfel J., Barkhof F. (2021). Quantitative magnetic resonance imaging towards clinical application in multiple sclerosis. Brain.

[14] Schmierer K., Scaravilli F., Altmann D.R., Barker G.J., Miller D.H. (2004). Magnetization transfer ratio and myelin in postmortem multiple sclerosis brain. Ann Neurol.

[15] Moccia M., van de Pavert S., Eshaghi A. (2020). Pathologic correlates of the magnetization transfer ratio in multiple sclerosis. Neurology.

[16] van Waesberghe J.H., Kamphorst W., De Groot C.J. (1999). Axonal loss in multiple sclerosis lesions: magnetic resonance imaging insights into substrates of disability. Ann Neurol.

[17] Johnson D., Ricciardi A., Brownlee W. (2021). Comparison of neurite orientation Dispersion and density imaging and two-compartment spherical mean technique parameter maps in multiple sclerosis. Front Neurol.

[18] van der Vuurst de Vries R.M., Mescheriakova J.Y., Wong Y.Y.M. (2018). Application of the 2017 revised McDonald criteria for multiple sclerosis to patients with a typical clinically isolated syndrome. JAMA Neurol.

[19] Hyun J.W., Kim W., Huh S.Y. (2019). Application of the 2017 McDonald diagnostic criteria for multiple sclerosis in Korean patients with clinically isolated syndrome. Mult Scler.

[20] Filippi M., Preziosa P., Meani A. (2022). Performance of the 2017 and 2010 revised McDonald criteria in predicting MS diagnosis after a clinically isolated syndrome: a MAGNIMS study. Neurology.

[21] Fadda G., Brown R.A., Longoni G. (2018). MRI and laboratory features and the performance of international criteria in the diagnosis of multiple sclerosis in children and adolescents: a prospective cohort study. Lancet Child Adolesc Health.

[22] Hacohen Y., Brownlee W., Mankad K. (2020). Improved performance of the 2017 McDonald criteria for diagnosis of multiple sclerosis in children in a real-life cohort. Mult Scler.

[23] Wong Y.Y.M., de Mol C.L., van der Vuurst de Vries R.M. (2019). Real-world validation of the 2017 McDonald criteria for pediatric MS. Neurol Neuroimmunol Neuroinflamm.

[24] Gaetani L., Prosperini L., Mancini A. (2018). 2017 Revisions of McDonald criteria shorten the time to diagnosis of multiple sclerosis in clinically isolated syndromes. J Neurol.

[25] Tintore M., Cobo-Calvo A., Carbonell P. (2021). Effect of changes in MS diagnostic criteria over 25 years on time to treatment and prognosis in patients with clinically isolated syndrome. Neurology.

[26] Poser C.M., Paty D.W., Scheinberg L. (1983). New diagnostic criteria for multiple sclerosis: guidelines for research protocols. Ann Neurol.

[27] Solomon A.J., Bourdette D.N., Cross A.H. (2016). The contemporary spectrum of multiple sclerosis misdiagnosis: a multicenter study. Neurology.

[28] Solomon A.J., Naismith R.T., Cross A.H. (2019). Misdiagnosis of multiple sclerosis: impact of the 2017 McDonald criteria on clinical practice. Neurology.

[29] Solomon A.J., Pettigrew R., Naismith R.T., Chahin S., Krieger S., Weinshenker B. (2021). Challenges in multiple sclerosis diagnosis: misunderstanding and misapplication of the McDonald criteria. Mult Scler.

[30] Wingerchuk D.M., Banwell B., Bennett J.L. (2015). International consensus diagnostic criteria for neuromyelitis optica spectrum disorders. Neurology.

[31] Banwell B., Bennett J.L., Marignier R. (2023). Diagnosis of myelin oligodendrocyte glycoprotein antibody-associated disease: international MOGAD panel proposed criteria. Lancet Neurol.

[32] Geraldes R., Jurynczyk M., Dos Passos G. (2020). Distinct influence of different vascular risk factors on white matter brain lesions in multiple sclerosis. J Neurol Neurosurg Psychiatry.

[33] Mainero C., Treaba C.A., Barbuti E. (2023). Imaging cortical lesions in multiple sclerosis. Curr Opin Neurol.

[34] Preziosa P., Rocca M.A., Mesaros S. (2018). Diagnosis of multiple sclerosis: a multicentre study to compare revised McDonald-2010 and Filippi-2010 criteria. J Neurol Neurosurg Psychiatry.

[35] Filippi M., Rocca M.A., Calabrese M. (2010). Intracortical lesions: relevance for new MRI diagnostic criteria for multiple sclerosis. Neurology.

[36] Cortese R., Prados Carrasco F., Tur C. (2023). Differentiating multiple sclerosis from AQP4-neuromyelitis optica spectrum disorder and MOG-antibody disease with imaging. Neurology.

[37] Seewann A., Kooi E.J., Roosendaal S.D. (2012). Postmortem verification of MS cortical lesion detection with 3D DIR. Neurology.

[38] Madsen M.A.J., Wiggermann V., Bramow S., Christensen J.R., Sellebjerg F., Siebner H.R. (2021). Imaging cortical multiple sclerosis lesions with ultra-high field MRI. Neuroimage Clin.

[39] Mainero C., Benner T., Radding A. (2009). In vivo imaging of cortical pathology in multiple sclerosis using ultra-high field MRI. Neurology.

[40] Muller J., La Rosa F., Beaumont J. (2022). Fluid and white matter suppression: new sensitive 3 T magnetic resonance imaging contrasts for cortical lesion detection in multiple sclerosis. Invest Radiol.

[41] Sati P., Oh J., Constable R.T. (2016). The central vein sign and its clinical evaluation for the diagnosis of multiple sclerosis: a consensus statement from the North American imaging in multiple sclerosis cooperative. Nat Rev Neurol.

[42] Haacke E.M., Mittal S., Wu Z., Neelavalli J., Cheng Y.C. (2009). Susceptibility-weighted imaging: technical aspects and clinical applications, part 1. AJNR Am J Neuroradiol.

[43] Castellaro M., Tamanti A., Pisani A.I., Pizzini F.B., Crescenzo F., Calabrese M. (2020). The use of the central vein sign in the diagnosis of multiple sclerosis: a systematic review and meta-analysis. Diagnostics (Basel).

[44] Daboul L., O'Donnell C.M., Cao Q. (2023). Effect of GBCA use on detection and diagnostic performance of the central vein sign: evaluation using a 3-T FLAIR∗ sequence in patients with suspected multiple sclerosis. AJR Am J Roentgenol.

[45] Maggi P., Absinta M., Grammatico M. (2018). Central vein sign differentiates multiple sclerosis from central nervous system inflammatory vasculopathies. Ann Neurol.

[46] Preziosa P., Rocca M.A., Filippi M. (2021). Central vein sign and iron rim in multiple sclerosis: ready for clinical use?. Curr Opin Neurol.

[47] Sinnecker T., Clarke M.A., Meier D. (2019). Evaluation of the central vein sign as a diagnostic imaging biomarker in multiple sclerosis. JAMA Neurol.

[48] Solomon A.J., Watts R., Ontaneda D., Absinta M., Sati P., Reich D.S. (2018). Diagnostic performance of central vein sign for multiple sclerosis with a simplified three-lesion algorithm. Mult Scler.

[49] Frischer J.M., Weigand S.D., Guo Y. (2015). Clinical and pathological insights into the dynamic nature of the white matter multiple sclerosis plaque. Ann Neurol.

[50] Clarke M.A., Pareto D., Pessini-Ferreira L. (2020). Value of 3T susceptibility-weighted imaging in the diagnosis of multiple sclerosis. AJNR Am J Neuroradiol.

[51] Maggi P., Sati P., Nair G. (2020). Paramagnetic rim lesions are specific to multiple sclerosis: an international multicenter 3T MRI study. Ann Neurol.

[52] Meaton I., Altokhis A., Allen C.M. (2022). Paramagnetic rims are a promising diagnostic imaging biomarker in multiple sclerosis. Mult Scler.

[53] Huang W., Sweeney E.M., Kaunzner U.W., Wang Y., Gauthier S.A., Nguyen T.D. (2022). Quantitative susceptibility mapping versus phase imaging to identify multiple sclerosis iron rim lesions with demyelination. J Neuroimaging.

[54] Chawla S., Kister I., Sinnecker T. (2018). Longitudinal study of multiple sclerosis lesions using ultra-high field (7T) multiparametric MR imaging. PLoS One.

[55] Kaunzner U.W., Kang Y., Zhang S. (2019). Quantitative susceptibility mapping identifies inflammation in a subset of chronic multiple sclerosis lesions. Brain.

[56] Tolaymat B., Zheng W., Chen H., Choi S., Li X., Harrison D.M. (2020). Sex-specific differences in rim appearance of multiple sclerosis lesions on quantitative susceptibility mapping. Mult Scler Relat Disord.

[57] Petzold A., Fraser C.L., Abegg M. (2022). Diagnosis and classification of optic neuritis. Lancet Neurol.

[58] Bennett J.L., Costello F., Chen J.J. (2023). Optic neuritis and autoimmune optic neuropathies: advances in diagnosis and treatment. Lancet Neurol.

[59] Biousse V., Danesh-Meyer H.V., Saindane A.M., Lamirel C., Newman N.J. (2022). Imaging of the optic nerve: technological advances and future prospects. Lancet Neurol.

[60] Dutra B.G., da Rocha A.J., Nunes R.H., Maia A.C.M.J. (2018). Neuromyelitis optica spectrum disorders: spectrum of MR imaging findings and their differential diagnosis. Radiographics.

[61] Hodel J., Outteryck O., Bocher A.L. (2014). Comparison of 3D double inversion recovery and 2D stir flair MR sequences for the imaging of optic neuritis: pilot study. Eur Radiol.

[62] Kupersmith M.J., Alban T., Zeiffer B., Lefton D. (2002). Contrast-enhanced MRI in acute optic neuritis: relationship to visual performance. Brain.

[63] Denis M., Woillez J.P., Smirnov V.M. (2022). Optic nerve lesion length at the acute phase of optic neuritis is predictive of retinal neuronal loss. Neurol Neuroimmunol Neuroinflamm.

[64] Outteryck O., Lopes R., Drumez E. (2020). Optical coherence tomography for detection of asymptomatic optic nerve lesions in clinically isolated syndrome. Neurology.

[65] Vidal-Jordana À., Rovira A., Calderon W. (2024). Adding the optic nerve in multiple sclerosis diagnostic criteria: a longitudinal, prospective, multicentre study. Neurology.

[66] Brownlee W.J., Miszkiel K.A., Tur C., Barkhof F., Miller D.H., Ciccarelli O. (2018). Inclusion of optic nerve involvement in dissemination in space criteria for multiple sclerosis. Neurology.

[67] Vidal-Jordana A., Rovira A., Arrambide G. (2021). Optic nerve topography in multiple sclerosis diagnosis: the utility of visual evoked potentials. Neurology.

[68] Bsteh G., Hegen H., Altmann P. (2023). Diagnostic performance of adding the optic nerve region assessed by optical coherence tomography to the diagnostic criteria for multiple sclerosis. Neurology.

[69] Bot J.C., Barkhof F. (2009). Spinal-cord MRI in multiple sclerosis: conventional and nonconventional MR techniques. Neuroimaging Clin N Am.

[70] Rocca M.A., Valsasina P., Meani A. (2023). Spinal cord lesions and brain grey matter atrophy independently predict clinical worsening in definite multiple sclerosis: a 5-year, multicentre study. J Neurol Neurosurg Psychiatry.

[71] Brownlee W.J., Altmann D.R., Prados F. (2019). Early imaging predictors of long-term outcomes in relapse-onset multiple sclerosis. Brain.

[72] Schlaeger R., Papinutto N., Panara V. (2014). Spinal cord gray matter atrophy correlates with multiple sclerosis disability. Ann Neurol.

[73] Westerlind H., Stawiarz L., Fink K., Hillert J., Manouchehrinia A. (2016). A significant decrease in diagnosis of primary progressive multiple sclerosis: a cohort study. Mult Scler.

[74] Shatila M., Ciccarelli O., Brownlee W. (2021). ECTRIMS 2021 – ePoster. Mult Sclerosis J.

[75] Elliott C., Belachew S., Wolinsky J.S. (2019). Chronic white matter lesion activity predicts clinical progression in primary progressive multiple sclerosis. Brain.

[76] Mahad D.H., Trapp B.D., Lassmann H. (2015). Pathological mechanisms in progressive multiple sclerosis. Lancet Neurol.

[77] Abdel-Aziz K., Schneider T., Solanky B.S. (2015). Evidence for early neurodegeneration in the cervical cord of patients with primary progressive multiple sclerosis. Brain.

[78] Aymerich F.X., Auger C., Alonso J. (2018). Cervical cord atrophy and long-term disease progression in patients with primary-progressive multiple sclerosis. AJNR Am J Neuroradiol.

[79] Tsagkas C., Magon S., Gaetano L. (2019). Preferential spinal cord volume loss in primary progressive multiple sclerosis. Mult Scler.

[80] Rocca M.A., Sormani M.P., Rovaris M. (2017). Long-term disability progression in primary progressive multiple sclerosis: a 15-year study. Brain.

[81] Paz Soldan M.M., Novotna M., Abou Zeid N. (2015). Relapses and disability accumulation in progressive multiple sclerosis. Neurology.

[82] Filippi M., Preziosa P., Barkhof F. (2021). Diagnosis of progressive multiple sclerosis from the imaging perspective: a review. JAMA Neurol.

[83] Braune S., Bluemich S., Bruns C. (2023). The natural history of primary progressive multiple sclerosis: insights from the German NeuroTransData registry. BMC Neurol.

[84] Confavreux C., Vukusic S. (2006). Natural history of multiple sclerosis: a unifying concept. Brain.

[85] Yan K., Balijepalli C., Desai K., Gullapalli L., Druyts E. (2020). Epidemiology of pediatric multiple sclerosis: a systematic literature review and meta-analysis. Mult Scler Relat Disord.

[86] Margoni M., Preziosa P., Rocca M.A., Filippi M. (2022). Pediatric multiple sclerosis: developments in timely diagnosis and prognostication. Expert Rev Neurother.

[87] Renoux C., Vukusic S., Mikaeloff Y. (2007). Natural history of multiple sclerosis with childhood onset. N Engl J Med.

[88] Gorman M.P., Healy B.C., Polgar-Turcsanyi M., Chitnis T. (2009). Increased relapse rate in pediatric-onset compared with adult-onset multiple sclerosis. Arch Neurol.

[89] McKay K.A., Hillert J., Manouchehrinia A. (2019). Long-term disability progression of pediatric-onset multiple sclerosis. Neurology.

[90] Baroncini D., Simone M., Iaffaldano P. (2021). Risk of persistent disability in patients with pediatric-onset multiple sclerosis. JAMA Neurol.

[91] Kopp T.I., Blinkenberg M., Chalmer T.A. (2020). Predictors of treatment outcome in patients with paediatric onset multiple sclerosis. Mult Scler.

[92] Capasso N., Virgilio E., Covelli A. (2023). Aging in multiple sclerosis: from childhood to old age, etiopathogenesis, and unmet needs: a narrative review. Front Neurol.

[93] Naseri A., Nasiri E., Sahraian M.A., Daneshvar S., Talebi M. (2021). Clinical features of late-onset multiple sclerosis: a systematic review and meta-analysis. Mult Scler Relat Disord.

[94] Vaughn C.B., Jakimovski D., Kavak K.S. (2019). Epidemiology and treatment of multiple sclerosis in elderly populations. Nat Rev Neurol.

[95] Mirmosayyeb O., Brand S., Barzegar M. (2020). Clinical characteristics and disability progression of early- and late-onset multiple sclerosis compared to adult-onset multiple sclerosis. J Clin Med.

[96] Alroughani R., Akhtar S., Ahmed S., Behbehani R., Al-Hashel J. (2016). Is time to reach EDSS 6.0 faster in patients with late-onset versus young-onset multiple sclerosis?. PLoS One.

[97] Kis B., Rumberg B., Berlit P. (2008). Clinical characteristics of patients with late-onset multiple sclerosis. J Neurol.

[98] Graves J.S., Krysko K.M., Hua L.H., Absinta M., Franklin R.J.M., Segal B.M. (2023). Ageing and multiple sclerosis. Lancet Neurol.

[99] Amato M.P., Fonderico M., Portaccio E. (2020). Disease-modifying drugs can reduce disability progression in relapsing multiple sclerosis. Brain.

[100] Jakimovski D., Kavak K.S., Vaughn C.B. (2022). Discontinuation of disease modifying therapies is associated with disability progression regardless of prior stable disease and age. Mult Scler Relat Disord.

[101] Lebrun-Frenay C., Kantarci O., Siva A. (2023). Radiologically isolated syndrome. Lancet Neurol.

[102] Okuda D.T., Siva A., Kantarci O. (2014). Radiologically isolated syndrome: 5-year risk for an initial clinical event. PLoS One.

[103] Lebrun-Frenay C., Kantarci O., Siva A. (2020). Radiologically isolated syndrome: 10-year risk estimate of a clinical event. Ann Neurol.

[104] Okuda D.T., Mowry E.M., Cree B.A. (2011). Asymptomatic spinal cord lesions predict disease progression in radiologically isolated syndrome. Neurology.

[105] Lebrun-Frenay C., Rollot F., Mondot L. (2021). Risk factors and time to clinical symptoms of multiple sclerosis among patients with radiologically isolated syndrome. JAMA Netw Open.

[106] Kantarci O.H., Lebrun C., Siva A. (2016). Primary progressive multiple sclerosis evolving from radiologically isolated syndrome. Ann Neurol.

[107] Okuda D.T., Mowry E.M., Beheshtian A. (2009). Incidental MRI anomalies suggestive of multiple sclerosis: the radiologically isolated syndrome. Neurology.

[108] De Stefano N., Giorgio A., Tintore M. (2018). Radiologically isolated syndrome or subclinical multiple sclerosis: MAGNIMS consensus recommendations. Mult Scler.

[109] Lebrun-Frenay C., Okuda D.T., Siva A. (2023). The radiologically isolated syndrome: revised diagnostic criteria. Brain.

[110] Fisniku L.K., Brex P.A., Altmann D.R. (2008). Disability and T2 MRI lesions: a 20-year follow-up of patients with relapse onset of multiple sclerosis. Brain.

[111] Tintore M., Rovira A., Rio J. (2015). Defining high, medium and low impact prognostic factors for developing multiple sclerosis. Brain.

[112] Minneboo A., Barkhof F., Polman C.H., Uitdehaag B.M., Knol D.L., Castelijns J.A. (2004). Infratentorial lesions predict long-term disability in patients with initial findings suggestive of multiple sclerosis. Arch Neurol.

[113] Tintore M., Rovira A., Arrambide G. (2010). Brainstem lesions in clinically isolated syndromes. Neurology.

[114] Chung K.K., Altmann D., Barkhof F. (2020). A 30-year clinical and magnetic resonance imaging observational study of multiple sclerosis and clinically isolated syndromes. Ann Neurol.

[115] Sombekke M.H., Wattjes M.P., Balk L.J. (2013). Spinal cord lesions in patients with clinically isolated syndrome: a powerful tool in diagnosis and prognosis. Neurology.

[116] Arrambide G., Rovira A., Sastre-Garriga J. (2018). Spinal cord lesions: a modest contributor to diagnosis in clinically isolated syndromes but a relevant prognostic factor. Mult Scler.

[117] Deloire M.S., Ruet A., Hamel D., Bonnet M., Dousset V., Brochet B. (2011). MRI predictors of cognitive outcome in early multiple sclerosis. Neurology.

[118] Ziccardi S., Pisani A.I., Schiavi G.M. (2023). Cortical lesions at diagnosis predict long-term cognitive impairment in multiple sclerosis: a 20-year study. Eur J Neurol.

[119] Summers M., Fisniku L., Anderson V., Miller D., Cipolotti L., Ron M. (2008). Cognitive impairment in relapsing-remitting multiple sclerosis can be predicted by imaging performed several years earlier. Mult Scler.

[120] Caramanos Z., Francis S.J., Narayanan S., Lapierre Y., Arnold D.L. (2012). Large, nonplateauing relationship between clinical disability and cerebral white matter lesion load in patients with multiple sclerosis. Arch Neurol.

[121] Ruggieri S., Prosperini L., Petracca M. (2023). The added value of spinal cord lesions to disability accrual in multiple sclerosis. J Neurol.

[122] Zecca C., Disanto G., Sormani M.P. (2016). Relevance of asymptomatic spinal MRI lesions in patients with multiple sclerosis. Mult Scler.

[123] Scalfari A., Romualdi C., Nicholas R.S. (2018). The cortical damage, early relapses, and onset of the progressive phase in multiple sclerosis. Neurology.

[124] Haider L., Prados F., Chung K. (2021). Cortical involvement determines impairment 30 years after a clinically isolated syndrome. Brain.

[125] Calabrese M., Filippi M., Rovaris M. (2009). Evidence for relative cortical sparing in benign multiple sclerosis: a longitudinal magnetic resonance imaging study. Mult Scler.

[126] Calabrese M., Romualdi C., Poretto V. (2013). The changing clinical course of multiple sclerosis: a matter of gray matter. Ann Neurol.

[127] Calabrese M., Rocca M.A., Atzori M. (2009). Cortical lesions in primary progressive multiple sclerosis: a 2-year longitudinal MR study. Neurology.

[128] Calabrese M., Rocca M.A., Atzori M. (2010). A 3-year magnetic resonance imaging study of cortical lesions in relapse-onset multiple sclerosis. Ann Neurol.

[129] Treaba C.A., Granberg T.E., Sormani M.P. (2019). Longitudinal characterization of cortical lesion development and evolution in multiple sclerosis with 7.0-T MRI. Radiology.

[130] Absinta M., Sati P., Masuzzo F. (2019). Association of chronic active multiple sclerosis lesions with disability in vivo. JAMA Neurol.

[131] Treaba C.A., Conti A., Klawiter E.C. (2021). Cortical and phase rim lesions on 7 T MRI as markers of multiple sclerosis disease progression. Brain Commun.

[132] Preziosa P., Filippi M., Rocca M.A. (2021). Chronic active lesions: a new MRI biomarker to monitor treatment effect in multiple sclerosis?. Expert Rev Neurother.

[133] Elliott C., Wolinsky J.S., Hauser S.L. (2019). Slowly expanding/evolving lesions as a magnetic resonance imaging marker of chronic active multiple sclerosis lesions. Mult Scler.

[134] Preziosa P., Pagani E., Meani A. (2022). Slowly expanding lesions predict 9-year multiple sclerosis disease progression. Neurol Neuroimmunol Neuroinflamm.

[135] Preziosa P., Pagani E., Moiola L., Rodegher M., Filippi M., Rocca M.A. (2021). Occurrence and microstructural features of slowly expanding lesions on fingolimod or natalizumab treatment in multiple sclerosis. Mult Scler.

[136] Calvi A., Carrasco F.P., Tur C. (2022). Association of slowly expanding lesions on MRI with disability in people with secondary progressive multiple sclerosis. Neurology.

[137] Calvi A., Tur C., Chard D. (2022). Slowly expanding lesions relate to persisting black-holes and clinical outcomes in relapse-onset multiple sclerosis. Neuroimage Clin.

[138] Elliott C., Arnold D.L., Chen H. (2020). Patterning chronic active demyelination in slowly expanding/evolving white matter MS lesions. AJNR Am J Neuroradiol.

[139] Beynon V., George I.C., Elliott C. (2022). Chronic lesion activity and disability progression in secondary progressive multiple sclerosis. BMJ Neurol Open.

[140] Calvi A., Clarke M.A., Prados F. (2023). Relationship between paramagnetic rim lesions and slowly expanding lesions in multiple sclerosis. Mult Scler.

[141] Haider L., Chung K., Birch G. (2021). Linear brain atrophy measures in multiple sclerosis and clinically isolated syndromes: a 30-year follow-up. J Neurol Neurosurg Psychiatry.

[142] Sastre-Garriga J., Pareto D., Battaglini M. (2020). MAGNIMS consensus recommendations on the use of brain and spinal cord atrophy measures in clinical practice. Nat Rev Neurol.

[143] Filippi M., Preziosa P., Copetti M. (2013). Gray matter damage predicts the accumulation of disability 13 years later in MS. Neurology.

[144] Rotstein D., Montalban X. (2019). Reaching an evidence-based prognosis for personalized treatment of multiple sclerosis. Nat Rev Neurol.

[145] Filippi M., Amato M.P., Centonze D. (2022). Early use of high-efficacy disease-modifying therapies makes the difference in people with multiple sclerosis: an expert opinion. J Neurol.

[146] Sormani M.P., Bruzzi P. (2013). MRI lesions as a surrogate for relapses in multiple sclerosis: a meta-analysis of randomised trials. Lancet Neurol.

[147] Sormani M.P., Arnold D.L., De Stefano N. (2014). Treatment effect on brain atrophy correlates with treatment effect on disability in multiple sclerosis. Ann Neurol.

[148] Gasperini C., Prosperini L., Tintore M. (2019). Unraveling treatment response in multiple sclerosis: a clinical and MRI challenge. Neurology.

[149] Rio J., Rovira A., Gasperini C. (2022). Treatment response scoring systems to assess long-term prognosis in self-injectable DMTs relapsing-remitting multiple sclerosis patients. J Neurol.

[150] Rio J., Castillo J., Rovira A. (2009). Measures in the first year of therapy predict the response to interferon beta in MS. Mult Scler.

[151] Sormani M.P., Rio J., Tintore M. (2013). Scoring treatment response in patients with relapsing multiple sclerosis. Mult Scler.

[152] Sormani M.P., Gasperini C., Romeo M. (2016). Assessing response to interferon-beta in a multicenter dataset of patients with MS. Neurology.

[153] Rotstein D.L., Healy B.C., Malik M.T., Chitnis T., Weiner H.L. (2015). Evaluation of no evidence of disease activity in a 7-year longitudinal multiple sclerosis cohort. JAMA Neurol.

[154] Kappos L., De Stefano N., Freedman M.S. (2016). Inclusion of brain volume loss in a revised measure of 'no evidence of disease activity' (NEDA-4) in relapsing-remitting multiple sclerosis. Mult Scler.

[155] Cacciaguerra L., Storelli L., Rocca M.A., Filippi M., Anitha S., Pillai B.M. (2022). Augmenting neurological disorder prediction and rehabilitation using artificial intelligence.

[156] Danelakis A., Theoharis T., Verganelakis D.A. (2018). Survey of automated multiple sclerosis lesion segmentation techniques on magnetic resonance imaging. Comput Med Imaging Graph.

[157] Garcia-Lorenzo D., Francis S., Narayanan S., Arnold D.L., Collins D.L. (2013). Review of automatic segmentation methods of multiple sclerosis white matter lesions on conventional magnetic resonance imaging. Med Image Anal.

[158] Afzal H.M.R., Luo S., Ramadan S., Lechner-Scott J. (2022). The emerging role of artificial intelligence in multiple sclerosis imaging. Mult Scler.

[159] La Rosa F., Abdulkadir A., Fartaria M.J. (2020). Multiple sclerosis cortical and WM lesion segmentation at 3T MRI: a deep learning method based on FLAIR and MP2RAGE. Neuroimage Clin.

[160] Schlaeger S., Shit S., Eichinger P. (2023). AI-based detection of contrast-enhancing MRI lesions in patients with multiple sclerosis. Insights Imaging.

[161] Dworkin J.D., Sati P., Solomon A. (2018). Automated integration of multimodal MRI for the probabilistic detection of the central vein sign in white matter lesions. AJNR Am J Neuroradiol.

[162] Maggi P., Fartaria M.J., Jorge J. (2020). CVSnet: a machine learning approach for automated central vein sign assessment in multiple sclerosis. NMR Biomed.

[163] Barquero G., La Rosa F., Kebiri H. (2020). RimNet: a deep 3D multimodal MRI architecture for paramagnetic rim lesion assessment in multiple sclerosis. Neuroimage Clin.

[164] Lou C., Sati P., Absinta M. (2020). Fully automated detection of paramagnetic rims in multiple sclerosis lesions on 3T susceptibility-based MR imaging. bioRxiv.

[165] Eitel F., Soehler E., Bellmann-Strobl J. (2019). Uncovering convolutional neural network decisions for diagnosing multiple sclerosis on conventional MRI using layer-wise relevance propagation. Neuroimage Clin.

[166] Lopatina A., Ropele S., Sibgatulin R., Reichenbach J.R., Gullmar D. (2020). Investigation of deep-learning-driven identification of multiple sclerosis patients based on susceptibility-weighted images using relevance analysis. Front Neurosci.

[167] Neeb H., Schenk J. (2019). Multivariate prediction of multiple sclerosis using robust quantitative MR-based image metrics. Z Med Phys.

[168] Sacca V., Sarica A., Novellino F. (2019). Evaluation of machine learning algorithms performance for the prediction of early multiple sclerosis from resting-state FMRI connectivity data. Brain Imaging Behav.

[169] Wang S.H., Tang C., Sun J. (2018). Multiple sclerosis identification by 14-layer convolutional neural network with batch normalization, dropout, and stochastic pooling. Front Neurosci.

[170] Yoo Y., Tang L.Y.W., Brosch T. (2018). Deep learning of joint myelin and T1w MRI features in normal-appearing brain tissue to distinguish between multiple sclerosis patients and healthy controls. Neuroimage Clin.

[171] Zurita M., Montalba C., Labbe T. (2018). Characterization of relapsing-remitting multiple sclerosis patients using support vector machine classifications of functional and diffusion MRI data. Neuroimage Clin.

[172] Eshaghi A., Wottschel V., Cortese R. (2016). Gray matter MRI differentiates neuromyelitis optica from multiple sclerosis using random forest. Neurology.

[173] Eshaghi A., Riyahi-Alam S., Saeedi R. (2015). Classification algorithms with multi-modal data fusion could accurately distinguish neuromyelitis optica from multiple sclerosis. Neuroimage Clin.

[174] Kim H., Lee Y., Kim Y.H. (2020). Deep learning-based method to differentiate neuromyelitis optica spectrum disorder from multiple sclerosis. Front Neurol.

[175] Rocca M.A., Anzalone N., Storelli L. (2021). Deep learning on conventional magnetic resonance imaging improves the diagnosis of multiple sclerosis mimics. Invest Radiol.

[176] Mangeat G., Ouellette R., Wabartha M. (2020). Machine learning and multiparametric brain MRI to differentiate hereditary diffuse leukodystrophy with spheroids from multiple sclerosis. J Neuroimaging.

[177] Theocharakis P., Glotsos D., Kalatzis I. (2009). Pattern recognition system for the discrimination of multiple sclerosis from cerebral microangiopathy lesions based on texture analysis of magnetic resonance images. Magn Reson Imaging.

[178] Bendfeldt K., Taschler B., Gaetano L. (2019). MRI-based prediction of conversion from clinically isolated syndrome to clinically definite multiple sclerosis using SVM and lesion geometry. Brain Imaging Behav.

[179] Wottschel V., Alexander D.C., Kwok P.P. (2015). Predicting outcome in clinically isolated syndrome using machine learning. Neuroimage Clin.

[180] Wottschel V., Chard D.T., Enzinger C. (2019). SVM recursive feature elimination analyses of structural brain MRI predicts near-term relapses in patients with clinically isolated syndromes suggestive of multiple sclerosis. Neuroimage Clin.

[181] Zhang H., Alberts E., Pongratz V. (2019). Predicting conversion from clinically isolated syndrome to multiple sclerosis-An imaging-based machine learning approach. Neuroimage Clin.

[182] Law M.T., Traboulsee A.L., Li D.K. (2019). Machine learning in secondary progressive multiple sclerosis: an improved predictive model for short-term disability progression. Mult Scler J Exp Transl Clin.

[183] Roca P., Attye A., Colas L. (2020). Artificial intelligence to predict clinical disability in patients with multiple sclerosis using FLAIR MRI. Diagn Interv Imaging.

[184] Zhao Y., Healy B.C., Rotstein D. (2017). Exploration of machine learning techniques in predicting multiple sclerosis disease course. PLoS One.

[185] Zhao Y., Wang T., Bove R. (2020). Ensemble learning predicts multiple sclerosis disease course in the SUMMIT study. NPJ Digit Med.

[186] Mendelsohn Z., Pemberton H.G., Gray J. (2023). Commercial volumetric MRI reporting tools in multiple sclerosis: a systematic review of the evidence. Neuroradiology.

